# Pathophysiological Effects and Targeted Therapy Strategies of Neutrophil Extracellular Traps in Ischemia–Reperfusion Injury

**DOI:** 10.3390/cells15171618

**Published:** 2026-09-05

**Authors:** Yan Lv, Linwu Kuang, Zhihan Xiao, Yingjie Zhang, Willice Wasonga Omindo, Xu Zhan, Xinji Liu, Qihang Sun, Yongyong Wang, Ruijie Zhang, Wei Ping, Qi Wang, Ni Zhang

**Affiliations:** 1 Department of Thoracic Surgery, Tongji Hospital, Tongji Medical College, Huazhong University of Science and Technology, 1095 Jie Fang Avenue, Wuhan 430030, China; lvyan020718@163.com (Y.L.); dockkk@163.com (L.K.); xiaozhihan1212@163.com (Z.X.); omindo2222@gmail.com (W.W.O.); zhanxu827@163.com (X.Z.); liuxinji1999@gmail.com (X.L.); lewen323@outlook.com (Q.S.); wangyongyong@tjh.tjmu.edu.cn (Y.W.); lmzrj@tjh.tjmu.edu.cn (R.Z.); pingwei@tjh.tjmu.edu.cn (W.P.); 2 Department of Histology and Embryology, School of Basic Medical Sciences, Tongji Medical College, Huazhong University of Science and Technology, Wuhan 430030, China; 18772866996@163.com

**Keywords:** ischemia–reperfusion injury, neutrophil extracellular traps, sterile inflammation, immunothrombosis, NETosis

## Abstract

Ischemia–reperfusion injury (IRI) is a common form of tissue injury encountered in myocardial infarction, ischemic stroke, solid-organ transplantation, and complex vascular surgery. Timely restoration of blood flow is essential for salvaging ischemic tissues; however, reperfusion itself can induce sterile inflammation and oxidative stress, further compromising microvascular and organ function. Accumulating evidence indicates that alterations in the local microenvironment associated with innate immune responses contribute to this pathological process, with neutrophils representing among the earliest effector cells recruited to injured tissues. In response to danger signals such as damage-associated molecular patterns, neutrophils can release neutrophil extracellular traps (NETs), extracellular web-like structures composed of decondensed chromatin and granular proteins. Extracellular NETs can injure endothelial and parenchymal cells and provide procoagulant scaffolds that contribute to immunothrombosis and local inflammatory responses. Current evidence is derived predominantly from clinical samples and experimental models across different organs. Owing to organ-specific differences in microvascular architecture, cellular composition, and ischemia–reperfusion conditions, the triggers, relative pathological contributions, and responses to NET-targeted interventions are not uniform across tissues. This review first summarizes the intracellular events preceding NET release, the molecular composition of extracellular NETs, and the mechanisms of NET extrusion. We then provide an organ-based synthesis of the local triggers, major injurious effects, and interventional evidence for NETs in the heart, liver, lung, kidney, brain, intestine, limb, and skin, while discussing recurrent pathological features—including microthrombosis, endothelial or barrier injury, and inflammatory amplification—in the context of organ-specific differences and the limitations of the available evidence. In addition, we evaluate therapeutic strategies involving degradation of extracellular NETs and neutralization of their toxic components, inhibition of NET-associated enzymes and upstream signaling pathways, and spatiotemporally targeted delivery, together with the major barriers to clinical translation. Overall, this review provides an organ-structured synthesis of current evidence linking NETs to IRI and offers a framework for understanding their context-dependent pathological roles and for developing organ- and phase-specific therapeutic strategies.

## 1. Introduction

Ischemia–reperfusion injury (IRI) represents a major clinical challenge in myocardial infarction, ischemic stroke, solid-organ transplantation, and complex vascular surgery and is closely associated with patient outcomes [1]. Timely restoration of blood flow is essential for salvaging ischemic tissues; however, reperfusion itself can induce secondary tissue injury [2]. Early studies of IRI focused primarily on intracellular events, including calcium overload, mitochondrial dysfunction, and excessive generation of reactive oxygen species [3,4]. Building on these findings, research in recent years has progressively expanded from parenchymal cell-intrinsic events to alterations within the tissue microenvironment, with dysregulation of the innate immune microenvironment and sterile inflammation increasingly recognized as contributors to IRI progression [5]. Among the immune cells involved, neutrophils are recruited relatively early to injured tissues after ischemia and participate in local inflammatory responses [6]. Since Brinkmann et al. first described neutrophil extracellular traps (NETs) in 2004, these web-like structures, composed of a decondensed chromatin scaffold decorated with multiple granular proteins such as neutrophil elastase, myeloperoxidase, and histones, were initially characterized as a mechanism of antimicrobial host defense but were subsequently implicated in a broad range of sterile inflammatory processes. Within the IRI microenvironment, increased NET formation or release, together with insufficient endogenous clearance, can increase the extracellular NET burden and may thereby exacerbate tissue injury [7,8].

From the perspective of the immune microenvironment, NETs can contribute to both parenchymal cell injury and microcirculatory dysfunction during IRI [9,10]. Cellular necrosis during ischemia and the early phase of reperfusion releases damage-associated molecular patterns (DAMPs) into the local microenvironment. These signals can activate neutrophils through pattern-recognition receptors such as Toll-like receptors (TLRs) and promote NET formation or release through mechanisms involving molecules such as peptidylarginine deiminase 4 (PAD4) [11,12]. Accumulated NETs can injure endothelial and parenchymal cells through NET-associated histones, while their web-like structures can entrap platelets and erythrocytes and thereby contribute to immunothrombosis [13,14]. In addition, NETs can modulate the inflammatory phenotypes of other innate immune cells, including macrophages, and interact with complement activation, thereby establishing a feedback loop in which inflammation and cellular injury reinforce each other [15,16]. Collectively, these processes may further compromise microvascular perfusion after reperfusion, contribute to the no-reflow phenomenon, and aggravate tissue damage [17,18]. Such mechanisms have been observed in studies of IRI affecting the heart, brain, liver, kidney, and other organs. However, their relative contributions, temporal profiles, and strength of supporting evidence vary across organs and experimental models. Accordingly, subsequent discussion should consider both recurrent mechanistic features and organ-specific differences [19].

In recent years, evidence implicating NETs in IRI has steadily accumulated. Nevertheless, the current evidence base remains dominated by preclinical studies, and substantial heterogeneity exists across organs with respect to ischemia duration, reperfusion time points, animal species, NET detection methods, and functional endpoints. Consequently, triggering pathways or therapeutic effects identified in one organ model cannot be directly extrapolated to other organs or clinical settings. Strategies aimed at degrading extracellular NETs or inhibiting NET formation and release have shown tissue-protective effects in multiple animal models; however, their clinical translation remains constrained by several factors, including potential impairment of host defense, risks of bleeding or secondary inflammatory responses, limited efficiency of lesion-targeted delivery, and the lack of specific dynamic biomarkers. Defining the predominant pathogenic processes, optimal therapeutic window, and acceptable safety margin within specific organs and disease contexts therefore remains an important unresolved issue. Against this background, existing evidence should be evaluated on an organ-by-organ basis, with particular attention to the local triggers of NETs, their tissue-damaging effects, therapeutic responses, and limitations of the available evidence in each form of organ IRI.

To address this heterogeneity in the evidence base and the associated challenges in clinical translation, this review summarizes the pathophysiological roles of NETs and NET-targeted therapeutic strategies across different forms of organ IRI. We first review the intracellular events preceding NET formation, the molecular composition of extracellular NETs, and the mechanisms through which NET components are released into the extracellular space. We then summarize, in sequence, the upstream triggers, major injurious effects, and interventional evidence within the local microenvironments of the heart, liver, lung, kidney, brain, intestine, limb, and skin, while retaining consideration of organ-specific differences in microcirculatory architecture, cellular interactions, and temporal disease dynamics. Finally, we evaluate the mechanistic levels targeted by current NET-directed strategies, the evidence supporting their therapeutic efficacy, their limitations, and the challenges associated with clinical translation. This review aims to provide an organ-structured synthesis of the available evidence and to identify pathological processes that recur across studies yet remain dependent on specific tissue contexts, thereby informing future organ-specific mechanistic investigations and therapeutic development.

## 2. NET Formation

**Figure 1:**
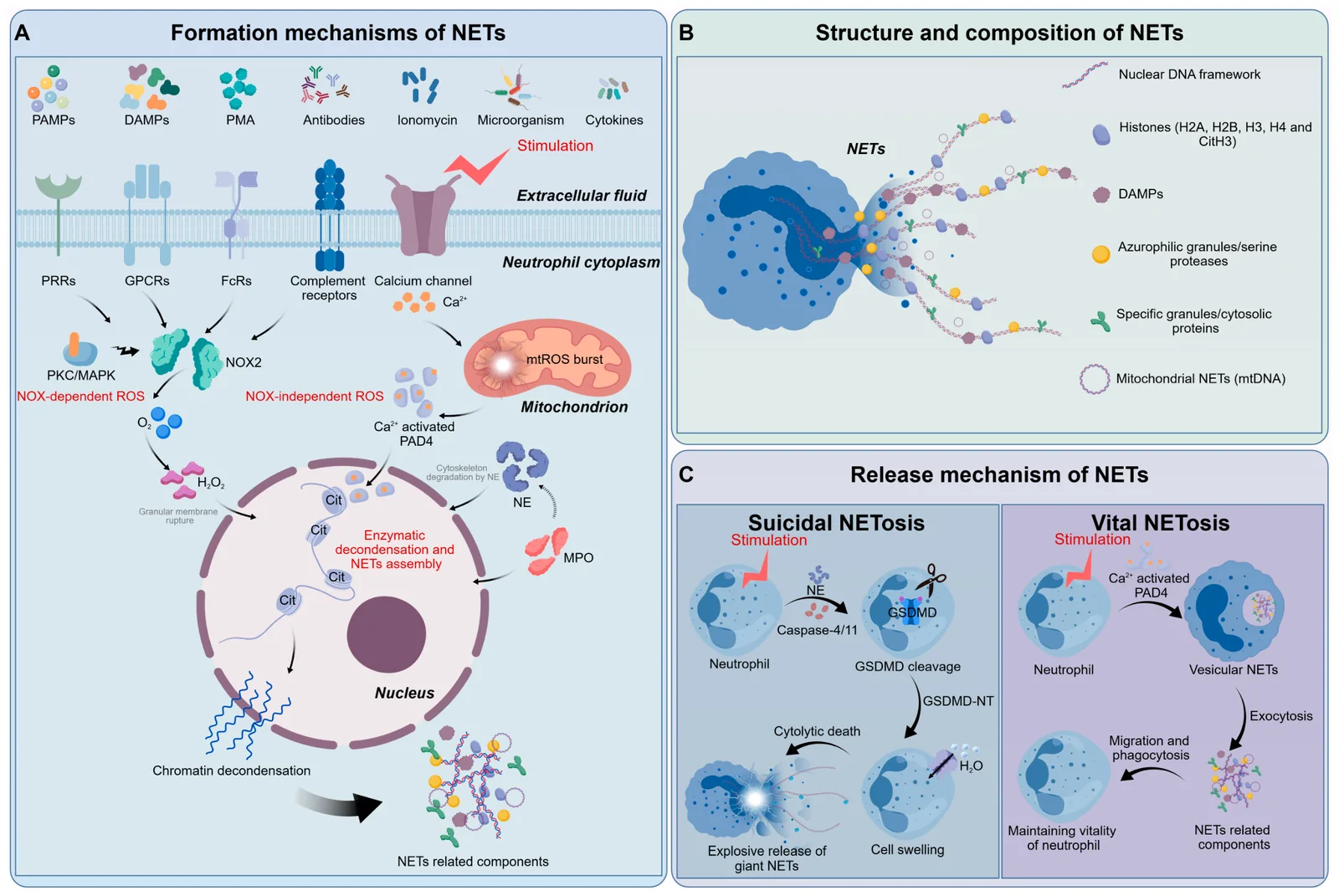
Intracellular mechanisms, extracellular composition, and modes of NET release. (**A**) Extracellular stimuli activate NOX-dependent or NOX-independent signaling through neutrophil receptors, promoting chromatin decondensation mediated by NE, MPO, and PAD4. (**B**) Extracellular NETs are composed of decondensed nuclear DNA or mtDNA scaffolds decorated with histones, granular proteins, and cytoplasmic proteins. (**C**) Lytic NETotic cell death can involve GSDMD-mediated increases in membrane permeability and subsequent cell rupture, whereas vital/non-lytic NET release extrudes chromatin through vesicular transport while preserving plasma membrane integrity and generating anucleate cytoplasts that retain transient effector functions.

Neutrophils are integral components of the innate immune system and participate in host defense against exogenous pathogens as well as in the mediation of sterile inflammation [20,21]. In addition to phagocytosis and degranulation, Brinkmann et al. reported in 2004 that, upon stimulation with agents such as phorbol 12-myristate 13-acetate (PMA), neutrophils can release web-like complexes composed of chromatin and multiple granular proteins, termed NETs [7]. Here, NETs are strictly defined as extracellular web-like structures composed of DNA/chromatin and neutrophil-derived proteins that have already been expelled into the extracellular space; intracellular decondensed chromatin or chromatin–granule protein precursor complexes that have not yet been externalized are not considered NETs. NET formation is used as an overarching process term encompassing stimulus sensing, chromatin decondensation, molecular mixing, and the eventual extrusion that generates extracellular NETs. NET release/extrusion refers specifically to the terminal step in which precursor complexes cross the cellular boundary and unfold extracellularly. The term NETosis is used only when evidence indicates that NET extrusion is accompanied by or results in neutrophil death. Processes in which neutrophils retain plasma membrane integrity and at least some effector functions are uniformly referred to here as vital/non-lytic NET release and are not classified as cell-death pathways [22]. Current evidence further indicates that NET formation contributes not only to antimicrobial defense but also to sterile inflammation, autoimmune disease, thrombosis, and the tumor microenvironment [8,16]. Release of extracellular traps is not unique to neutrophils; other innate immune cells, including mast cells, eosinophils, basophils, and macrophages, can generate analogous structures under specific stimuli [23,24]. NET formation is regulated by multilayered signaling networks and is spatially constrained, involving receptor activation, cytoskeletal remodeling, reactive oxygen species generation, epigenetic modification, and transmembrane trafficking or membrane pore formation [25,26]. The molecular and cellular mechanisms underlying this process are summarized below from three perspectives: intracellular events preceding NET release, the major structural components of extracellular NETs, and the modes of NET release (Figure 1).

### 2.1. Intracellular Events Preceding NET Release

Within this sequence of events, stimulus sensing and Ca^2+^ mobilization represent relatively early intracellular steps. NET formation can be initiated by a series of regulated intracellular events [27]. Pattern recognition receptors (PRRs), G protein-coupled receptors (GPCRs), Fc receptors (FcRs), and complement receptors on the neutrophil surface can recognize diverse physiological and pathological ligands [28,29]. These ligands include pathogen-associated molecular patterns (PAMPs), endogenous damage-associated molecular patterns (DAMPs) such as high mobility group box 1 (HMGB1) and cell-free DNA (cfDNA), as well as experimental inducers including PMA, selected antibodies, and calcium ionophores such as A23187 and ionomycin [30,31]. Receptor activation can induce fluctuations in intracellular calcium (Ca^2+^) levels [32]. This calcium signal originates from mobilization of endoplasmic reticulum Ca^2+^ stores and can act in concert with store-operated calcium entry mediated by stromal interaction molecule 1 (STIM1) and calcium release-activated calcium modulator 1 (ORAI1) channels, thereby increasing cytosolic Ca^2+^ concentrations [33]. Elevated cytosolic Ca^2+^ functions as a second messenger that activates protein kinase C (PKC) and mitogen-activated protein kinase (MAPK) cascades, including the rapidly accelerated fibrosarcoma (Raf)–mitogen-activated protein kinase (MEK)–extracellular signal-regulated kinase (ERK) pathway, while also creating conditions permissive for the subsequent translocation and activation of relevant enzymes [34,35].

These Ca^2+^ signals can couple to cellular oxidative systems, although the principal source of reactive oxygen species (ROS) depends on the nature of the stimulus. ROS generation can contribute to chromatin decondensation and alterations in nuclear-envelope integrity [36]. In PMA-induced lytic NETotic cell death, traditionally termed suicidal NETosis, activation of PKC and MAPK pathways promotes translocation of cytosolic nicotinamide adenine dinucleotide phosphate (NADPH) oxidase subunits, such as p47^phox, and their assembly with membrane-associated subunits such as gp91^phox on the plasma or granule membrane to form an active NADPH oxidase 2 (NOX2) complex [37]. NOX2 catalyzes the reduction of molecular oxygen to superoxide anion, which is subsequently converted by superoxide dismutase (SOD) into hydrogen peroxide (H_2_O_2_), constituting part of the neutrophil respiratory burst [38]. Accumulated H_2_O_2_ can compromise granule-membrane integrity, allowing granular constituents to enter the cytosol [39]. In NOX-independent pathways induced by calcium ionophores or specific microcrystals, such as cholesterol crystals, elevated cytosolic Ca^2+^ can enter the mitochondrial matrix, promote opening of the mitochondrial permeability transition pore, perturb the electron transport chain, and increase mitochondrial reactive oxygen species (mtROS) generation, thereby providing oxidative signals for downstream events [40,41].

Irrespective of whether ROS are derived predominantly from NOX2 or mitochondria, their downstream effects can converge on granule-enzyme release and chromatin decondensation. As granule-membrane integrity declines, nuclear translocation and activation of neutrophil enzymes contribute to the decondensation of nuclear chromatin [42]. Under resting conditions, PAD4, a Ca^2+^-dependent hydrolase, exhibits relatively low activity because cytosolic Ca^2+^ concentrations remain low [43]. When cytosolic Ca^2+^ levels rise, Ca^2+^ binding to multiple sites on PAD4 induces conformational changes in its catalytic domain and facilitates enzyme activation [44,45]. Activated PAD4 can translocate to the nucleus, where it catalyzes deimination of arginine residues on proteins such as histones H3 and H4, converting them into citrulline residues [46,47]. This modification reduces the positive charge of histones and weakens their electrostatic interactions with the negatively charged phosphate backbone of DNA, thereby promoting nucleosomal relaxation [48]. In parallel, neutrophil elastase (NE), myeloperoxidase (MPO), and other granular constituents can be released into the cytosol. NE can bind to and degrade filamentous actin, thereby restricting cytoskeletal remodeling as well as neutrophil migration and polarization [36,39]. NE and MPO can subsequently translocate into the nucleus; NE degrades histones, whereas MPO can use H_2_O_2_ to participate in intranuclear protein modification and perinuclear lipid oxidation [36,49]. Through the combined effects of these enzymatic and oxidative events, chromatin progressively decondenses, the nucleus swells and loses its characteristic multilobulated morphology, and declining nuclear-envelope integrity allows decondensed chromatin to diffuse into the cytoplasm and mix with granular proteins, generating chromatin–granule protein complexes poised for extracellular release [22,25].

### 2.2. Structure and Major Components of Extracellular NETs

Following extrusion of chromatin–granule protein complexes, the extracellular architecture and molecular composition of NETs can influence their subsequent biological effects. Ultrastructural studies have described NETs as three-dimensional meshes composed of double-stranded DNA fibers approximately 15–17 nm in diameter, decorated with globular protein domains approximately 25 nm in diameter, together forming pores of approximately 200 nm [7,50]. These structures possess recognizable physical and immunological characteristics, although their molecular composition is not invariant [16]. High-resolution mass spectrometry-based proteomic studies have demonstrated compositional heterogeneity among NETs induced by different stimuli, including PMA, lipopolysaccharide (LPS), and calcium ionophores. Collectively, these studies have identified approximately 330 proteins and reported a shared core comprising several dozen proteins. According to their subcellular origins, these constituents can broadly be classified as nuclear proteins, granular proteins, and other cytoplasmic components [51,52].

Within this three-dimensional architecture, nucleic acids and nuclear proteins can account for more than 70% of the total NET protein mass. The principal physical scaffold consists of decondensed double-stranded nuclear DNA (nDNA) associated with positively charged histones [53]. Core histones include H2A, H2B, H3, and H4. In certain NET-forming pathways, PAD4-mediated citrullinated histone H3 (CitH3) is commonly used as an associated marker; however, detection of CitH3 alone is insufficient to establish the presence of extracellular NETs and should preferably be combined with evidence of colocalization or complex formation between extracellular DNA and neutrophil proteins such as MPO or NE [54,55]. HMGB1 bound to DNA fibers can function, following NET release, as a pro-inflammatory DAMP that modulates neighboring immune cells [56]. Enzymes derived from azurophilic and specific granules confer local proteolytic, oxidative, and cytotoxic activities on NETs [13]. Azurophilic granule constituents include NE, MPO, proteinase 3 (PR3), cathepsin G, and other serine proteases and oxidases, some of which retain enzymatic activity on the NET surface [57]. Proteins derived from specific granules and the cytoplasm include lactoferrin, the antimicrobial peptide LL-37, matrix metalloproteinase-9 (MMP9), and the calprotectin complex formed by S100A8 and S100A9. Among these, LL-37 can interact with cellular membranes and increase membrane permeability, whereas S100A8/A9 can enhance local immune responses through the TLR4/myeloid differentiation primary response 88 (MYD88) signaling axis [58,59].

Although the NETs described above primarily use nuclear double-stranded DNA as their scaffold, the nucleic-acid origin of extracellular traps is not uniform. In addition to nDNA, NETs may contain mitochondrial DNA (mtDNA), reflecting differences between nuclear- and mitochondrial-derived extracellular traps [60]. Under specific pathological stresses, severe trauma, or conditions involving granulocyte–macrophage colony-stimulating factor (GM-CSF) priming followed by complement component 5a (C5a) or LPS stimulation, neutrophils can release mitochondrial DNA extracellularly through a process that does not involve cell lysis or nuclear rupture, generating histone-poor web-like structures referred to as mitochondrial NETs [60,61]. In contrast to histone-coated nDNA, mtDNA lacks histone protection and contains unmethylated CpG motifs and oxidative modifications [41,62]. These features allow mtDNA to activate double-stranded DNA-sensing pathways, including cyclic GMP–AMP synthase (cGAS)–stimulator of interferon genes (STING) and endosomal TLR9, thereby inducing the expression of type I interferons and pro-inflammatory mediators [41,63].

### 2.3. Modes of NET Release

Whereas structure and composition define the extracellular entity of NETs, the temporal characteristics of their biological effects are also influenced by how these complexes cross the cellular boundary. Decondensed chromatin within neutrophils can mix with granular and cytoplasmic proteins before extrusion; however, these materials are termed NETs only after they have crossed the cellular boundary and unfolded in the extracellular space. Based on the eventual state of the plasma membrane, release kinetics, and execution mechanisms, NET extrusion can broadly be divided into lytic NETotic cell death and vital/non-lytic NET release [64]. Lytic NETotic cell death, also referred to as suicidal NETosis, generally proceeds over a relatively prolonged period, commonly 3–8 h after stimulation, and is characterized by loss of plasma-membrane integrity, NET extrusion, and lytic cell death [22]. Frequently used experimental inducers include PMA, cholesterol crystals, selected immune complexes, and high concentrations of autoantibodies [16,65]. Under certain conditions, the pore-forming protein gasdermin D (GSDMD) contributes to changes in membrane permeability and chromatin extrusion. Following stimulation, NE released into the cytosol or inflammatory caspases activated through the noncanonical inflammasome pathway, such as caspase-4/11, can cleave GSDMD at specific sites to generate a pore-forming N-terminal fragment (GSDMD-NT) [26,66]. GSDMD-NT can oligomerize and bind phospholipids in the nuclear and plasma membranes, forming transmembrane pores. These pores can compromise nuclear- and plasma-membrane integrity, facilitate movement of decondensed chromatin into the cytoplasm, and promote osmotic cell swelling [67,68]. Subsequent rupture of the plasma membrane permits extrusion of chromatin–protein complexes, which then unfold extracellularly to form NETs [25].

In contrast to lytic NETotic cell death, vital/non-lytic NET release, historically also termed vital NETosis, does not involve immediate lytic cell death and can occur within 5–60 min after stimulation [69]. This process can be triggered by specific pathogens, such as Staphylococcus aureus through TLR2 and Escherichia coli through TLR4, or by signals associated with activated platelets. Under certain experimental conditions, this form of NET release depends on Ca^2+^-associated PAD4 activity and may show less dependence on NOX2-mediated cytosolic ROS generation [64,70]. During vital NET release, following chromatin decondensation, the inner and outer nuclear membranes can separate and reorganize, packaging DNA together with associated granular enzymes into vesicle-like structures through a budding-like process [27,69]. These vesicles can subsequently be transported toward the plasma membrane along microtubule and actin cytoskeletal networks, after which chromatin–protein complexes are extruded through an exocytosis-like process and expand extracellularly to form NETs, while plasma-membrane integrity is maintained or restored [8]. This non-lytic mode of release can leave behind anucleate but temporarily viable neutrophil cytoplasts. Studies have reported that these cytoplasts can retain membrane potential for a period of time and preserve functions including chemotactic migration, amoeboid motility, and phagocytosis [71]. Thus, non-lytic NET release may generate extracellular trapping structures while preserving a subset of neutrophil effector functions [20]. Although both lytic NETotic cell death and non-lytic NET release increase the extracellular NET burden, they may generate distinct cellular remnants and inflammatory consequences.

## 3. Roles of NETs in IRI Across Different Organs

**Figure 2:**
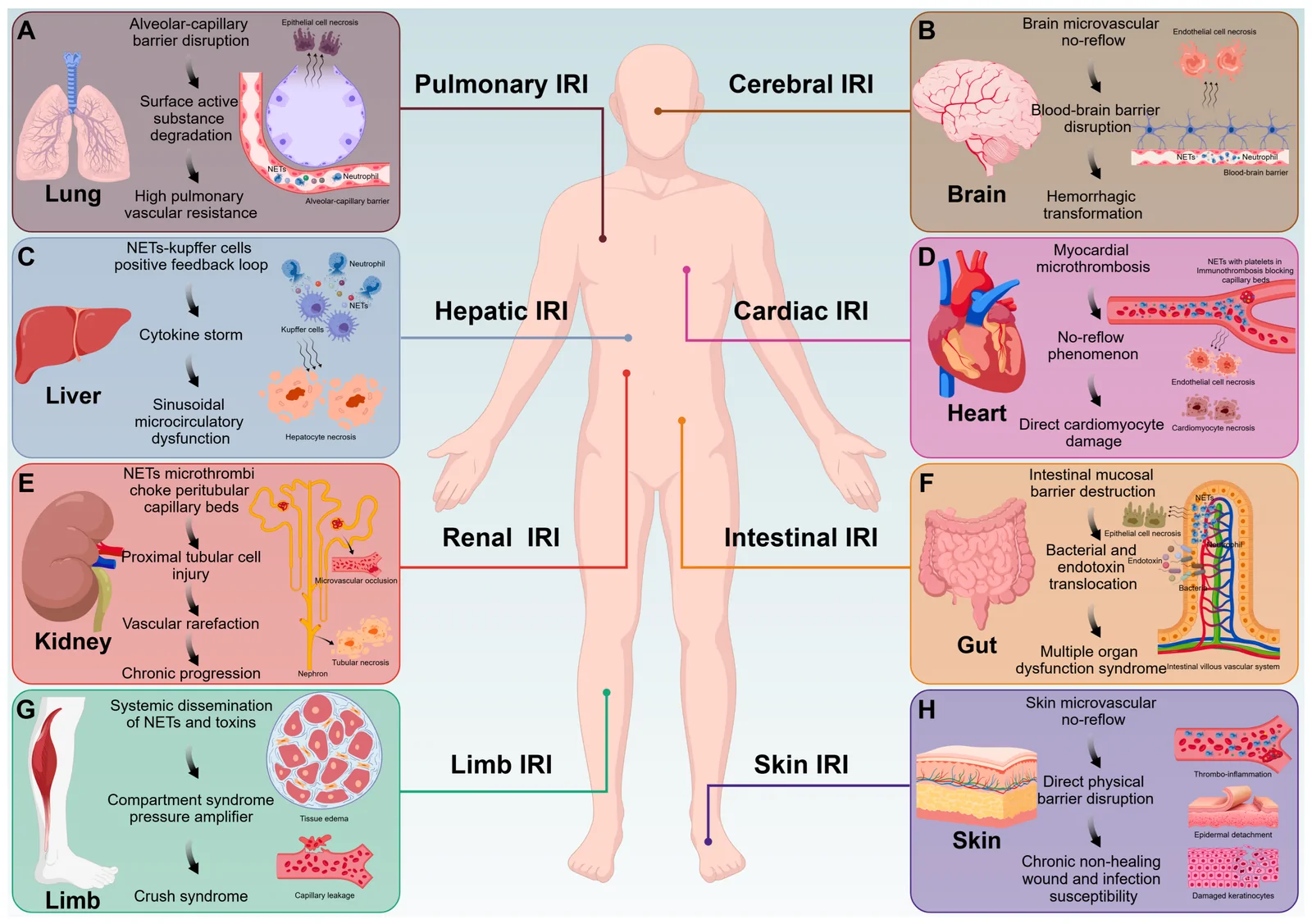
Pathological roles of extracellular NETs in IRI across different organs. (**A**) Pulmonary IRI: alveolar epithelial and alveolar–capillary barrier injury, surfactant dysfunction, and increased pulmonary vascular resistance. (**B**) Cerebral IRI: microvascular no-reflow, endothelial and BBB injury, and an increased risk of hemorrhagic transformation. (**C**) Hepatic IRI: pro-inflammatory interactions with Kupffer cells, hepatic sinusoidal microcirculatory dysfunction, and hepatocellular injury. (**D**) Cardiac IRI: NET-associated immunothrombosis, microcirculatory obstruction, and injury to cardiomyocytes and endothelial cells. (**E**) Renal IRI: peritubular capillary obstruction, tubular injury, and progression from acute kidney injury to chronic kidney disease. (**F**) Intestinal IRI: villous microcirculatory and epithelial barrier injury, microbial translocation, and amplification of systemic inflammation. (**G**) Limb IRI: local edema and increased compartment pressure, together with remote-organ injury mediated by circulating NET-associated components. (**H**) Skin IRI: thromboinflammation, microvascular no-reflow, barrier disruption, and delayed wound healing.

Increased NET formation or extracellular NET burden has been observed in IRI across multiple organs; however, the triggering signals, principal effector cells, pathological consequences, and temporal dynamics are strongly influenced by the local organ microenvironment [19,72]. An organ-specific overview is provided in Figure 2. The heart, liver, lung, kidney, brain, intestine, limb, and skin differ substantially in microvascular architecture, hemodynamics, parenchymal cell metabolism, and the composition of resident immune cells. Therefore, NETs cannot be presumed to follow an identical pathogenic pathway across all organs. Accordingly, we adopt an organ-based structure to summarize the local triggers, major tissue-damaging effects, and reported interventional outcomes in each form of IRI, with the corresponding evidence summarized in Table 1.

### 3.1. NETs and Cardiac IRI

Studies of cardiac IRI suggest that NETs may contribute to the link between immune inflammation and microcirculatory no-reflow. Although early reperfusion is the standard treatment for acute myocardial infarction, myocardial IRI can still be accompanied by arrhythmias, myocardial stunning, and no-reflow, thereby limiting the benefits of revascularization [1,4,155,156,157,158]. In this setting, neutrophil infiltration and increased NET release are considered among the processes associated with myocardial injury [10]. Several studies have shown that metabolic and transcriptional alterations within the myocardial microenvironment can regulate NET formation. For example, peripheral blood neutrophils from patients with IRI may exhibit an MMP9-high phenotype associated with the SPI1/CST7 axis and accompanied by increased NET formation [73]. Lactate accumulation in ischemic regions can also induce histone H3K18 lactylation, thereby affecting MICU3 transcription, mitochondria-associated endoplasmic reticulum membranes (MAMs), and calcium homeostasis and ultimately promoting NET formation and release [74]. In addition, endoplasmic reticulum stress mediated by the microsomal glutathione S-transferase 2 (MGST2)/leukotriene C4 (LTC4)/NOX2 pathway in the context of aldehyde dehydrogenase 2 (ALDH2) deficiency, as well as gut microbiota translocation, has been associated with NET formation and extracellular accumulation during myocardial IRI [75,76,159].

Among the reported downstream effects, NETs can serve as a physical scaffold for microvascular thrombosis and may thereby contribute to microcirculatory obstruction and no-reflow. Accumulated NETs can induce dysfunction and apoptosis of cardiac microvascular endothelial cells, while their DNA networks and histones can interact with activated platelets and coagulation factors [74,76]. Mechanistic studies have shown that NETs can promote platelet activation through platelet cluster of differentiation 36 (CD36)-mediated extracellular signal-regulated kinase 5 (ERK5) signaling and become intertwined with von Willebrand factor (VWF)-rich microthrombi, thereby increasing microvascular embolization [10,77]. Targeting this process, degradation of the NET scaffold has shown potential to improve reperfusion in experimental studies. Early administration of deoxyribonuclease I (DNase I) after reperfusion, either alone or in combination with recombinant human a disintegrin and metalloproteinase with thrombospondin type 1 motif, member 13 (ADAMTS13), can reduce extracellular chromatin in ischemic regions; when combined with recombinant tissue plasminogen activator (rt-PA), it can also improve endpoints related to thrombolysis, no-reflow, and left ventricular remodeling [10,17]. In addition, colchicine, the interleukin-6 (IL-6) receptor inhibitor tocilizumab, and mesenchymal stem cell (MSC)-derived exosomes have each been associated with reductions in microvascular obstruction or myocardial infarct size in corresponding experimental models [78,79,80].

Beyond physical vascular obstruction, NETs can also participate in intercellular inflammatory signaling within the myocardium. During reperfusion, infiltrating M1-like macrophages can activate the p38 MAPK pathway through the thrombospondin 1 (THBS1)/cluster of differentiation 47 (CD47) axis and promote NET release by neutrophils; 4-octyl itaconate (4-OI) has been shown to suppress this process in corresponding models [81]. Activation of the tumor necrosis factor-related apoptosis-inducing ligand (TRAIL)–death receptor 5 (DR5) pathway has also been associated with cardiomyocyte apoptosis, neutrophil chemotaxis, and increased NET formation [82,160,161,162]. The resulting mutually reinforcing feedback may further impair mitochondrial membrane potential and cell survival. Accordingly, both inhibition of NET formation or release and neutralization of NET-associated toxic components have been explored as experimental cardioprotective strategies. Genetic deletion of Padi4 or pharmacological PAD4 inhibition can preserve myocardial mitochondrial integrity and reduce apoptosis in certain models [10,83]. Early administration of a neutralizing antibody against citrullinated histone H3 (hCitH3-mAb) after reperfusion has also been associated with reduced infarct size [84]. Furthermore, exploiting the ability of neutrophils to migrate toward injured myocardium, investigators have developed cell-mediated biomimetic nanodelivery systems to deliver sarcoplasmic reticulum (SR) calcium-pump activators to myocardial lesions, with improved cardiac remodeling observed in animal models [85]. These studies provide experimental support for localized delivery strategies, although their selectivity, safety, and clinical applicability require further evaluation. The coexistence of microthrombosis and inflammation observed in cardiac IRI provides a useful reference for comparison with other organs.

### 3.2. NETs and Hepatic IRI

In contrast to cardiac IRI, in which research has focused predominantly on coronary microcirculation and no-reflow, studies of hepatic IRI additionally address the relationship between the hepatic sinusoidal immune ecosystem and surgical outcomes. Hepatic IRI can occur during liver resection, orthotopic liver transplantation, and traumatic hemorrhagic shock. The liver possesses a low-pressure, high-capacitance microcirculatory system and contains a substantial pool of marginated neutrophils within the vascular compartment; consequently, ischemic stress can provoke sterile inflammation and microthrombosis [163,164,165]. Studies indicate that during the early phase of hepatic IRI, neutrophil recruitment, NET release, and extracellular NET accumulation are coordinately regulated by signals from multiple cell types within the hepatic sinusoids. On the one hand, damage-associated molecular patterns (DAMPs), such as HMGB1, and superoxide released from injured hepatocytes and Kupffer cells can promote NET formation and release through TLR4- and NOX-related pathways [11,86,166]. On the other hand, interleukin-33 (IL-33) released by liver sinusoidal endothelial cells can interact with suppression of tumorigenicity 2 (ST2) receptors on neutrophils and promote NET formation [87]. Complement signaling involving complement component 5a receptor 1 (C5aR1), macrophage–neutrophil interactions, and ferroptotic pathways associated with the E3 ubiquitin ligase tripartite motif-containing 21 (TRIM21) have also been implicated in the regulation of NET formation [88,89,167,168,169]. The liver simultaneously possesses endogenous mechanisms that restrain NET formation; for example, histidine-rich glycoprotein (HRG) and the long isoform of carcinoembryonic antigen-related cell adhesion molecule 1 (CC1-L) on the neutrophil surface can suppress NET formation through mechanisms involving autophagy [90,91]. Together, these molecular pathways influence the extracellular NET burden and its tissue effects during hepatic IRI.

In addition to local inflammation and sinusoidal microcirculatory injury, hepatic studies suggest that IRI-associated NETs may influence tumor recurrence and metastasis. In hepatic surgical models involving malignancy, serum/glucocorticoid-regulated kinase 1 (SGK1) expressed by hepatic parenchymal cells can activate the IL-6/signal transducer and activator of transcription 3 (STAT3)/serum amyloid A (SAA) axis, promoting the recruitment of polymorphonuclear myeloid-derived suppressor cells (PMN-MDSCs) and NET release and thereby generating a local environment that may favor colorectal cancer liver metastasis [92,170,171]. Spleen tyrosine kinase (SYK) activation can also promote macrophage release of IL-1β and enhance NET formation through nuclear translocation of pyruvate kinase M2 (PKM2) in neutrophils; these changes have been associated with increased postoperative recurrence of hepatocellular carcinoma in corresponding models [93,172,173]. In addition, NETs entering the circulation during surgical stress can interact with activated platelets through an ERK5–platelet glycoprotein IIb/IIIa integrin-related mechanism, capture circulating tumor cells (CTCs), and form microemboli, potentially facilitating pulmonary metastasis [94].

Based on experimental observations involving both local tissue injury and oncological outcomes, inhibition of NET formation or release has been investigated as a candidate strategy for improving outcomes after hepatic surgery. Recombinant human thrombomodulin (rTM) can suppress TLR4 and its downstream MAPK/ROS/PAD4 signaling and has been shown to reduce NET formation and hepatocyte apoptosis in corresponding models [95]. Hydroxychloroquine (HCQ) can also exert hepatoprotective effects by modulating the TLR9/PAD4 pathway [96]. In marginal donor liver models such as steatotic liver grafts, simvastatin attenuates hyperlipidemia-associated neutrophil activation and NET release by downregulating the oxidized low-density lipoprotein (oxLDL)/macrophage-1 antigen (Mac-1) axis [97]. Polyphenolic natural products such as curcumin and tetramethylpyrazine (TMP) have also been reported to reduce NET formation by modulating NADPH oxidase- and MEK/ERK-related pathways [98,99,174,175,176]. Regular exercise training, as a non-pharmacological preconditioning strategy, can alter the hepatic immune-cell composition and has been associated in animal studies with increased M2-like macrophages and antitumor T cells together with a reduced extracellular NET burden [100]. These findings are derived predominantly from preclinical studies; future clinical investigations will need to evaluate the balance among lesion-targeted delivery, control of NET-associated injury, and preservation of systemic antimicrobial host defense.

### 3.3. NETs and Pulmonary IRI

Pulmonary IRI is associated with primary graft dysfunction (PGD) and adverse early outcomes after lung transplantation [177,178,179]. Cold ischemia and hilar clamping can induce sterile pulmonary inflammation accompanied by neutrophil extravasation and NET accumulation [180]. Research on pulmonary IRI has placed particular emphasis on the relationship between intracellular mitochondrial homeostasis and NET formation. Mitochondrial DNA (mtDNA) released under ischemic stress can function as an endogenous danger signal, promoting NET release and extracellular NET accumulation through the TLR9 pathway [101]. This process is further regulated by multiple protein networks. Upregulation of the mitochondrial matrix peptidyl-prolyl cis-trans isomerase F (PPIF) can increase calcium loading and mitochondrial oxidative stress and promote NET formation through the calcineurin (CN)/nuclear factor of activated T cells (NFAT) pathway [102]. Conversely, one study showed that following blockade of the pore-forming protein GSDMD, mtDNA could translocate into the neutrophil cytoplasm and activate the cGAS–STING pathway, thereby establishing a compensatory signal that sustains NET release [103]. This finding suggests that, in the corresponding pulmonary IRI model, inhibition of membrane-pore formation alone may be insufficient to block mtDNA-associated NET formation.

At the tissue level, the extracellular NET burden during pulmonary IRI is regulated by interactions among endothelial cells, alveolar macrophages (AMs), and neutrophils. First, upregulation of the arachidonate 12-lipoxygenase (ALOX12)/12-hydroxyeicosatetraenoic acid (12-HETE) axis during ischemia can promote endothelial ferroptosis and HMGB1 release, thereby enhancing NET formation and release through neutrophil TLR4/MYD88 signaling [104]. Activation of endothelial TLR4/NOX4 signaling can additionally contribute to neutrophil migration into subpleural regions and alveolar spaces [181]. Second, macrophage-derived extracellular vesicles (EVs) can deliver endothelial monocyte-activating polypeptide II (EMAP-II) to neutrophils, increasing mitochondrial ROS generation and promoting NET formation through inhibition of the phosphoinositide 3-kinase (PI3K)/protein kinase B (AKT) pathway [105]. Conversely, NET-associated extracellular histones can drive AMs toward a pro-inflammatory phenotype, increase lysosomal membrane permeabilization (LMP), and promote cathepsin C (CTSC) release. CTSC can then act on neutrophils through the ROS/p38 MAPK/p47^phox pathway to enhance NADPH oxidase activity, thereby establishing a mutually reinforcing feedback loop between NETs and macrophages [106].

On the basis of these intracellular triggers and intercellular feedback mechanisms, interventions targeting PPIF, the ALOX12 inhibitor ML355, EMAP-II, or CTSC have each been associated with improved oxygenation and reduced pulmonary tissue injury in corresponding models [102,104,105,106]. However, in the translational context of lung transplantation, direct NET degradation also entails immunological trade-offs. Intratracheal or intravenous DNase I can reduce pulmonary NET structures and improve early respiratory function, but NET fragments generated by degradation may retain immunostimulatory activity [180,181]. These fragments can activate TLR–MYD88 signaling in AMs and sustain local inflammation, while also promoting dendritic-cell maturation and expansion of alloantigen-specific CD4+ T cells, potentially compromising immune tolerance after lung transplantation [107]. Thus, therapeutic evaluation in lung transplant-associated IRI should not be limited to degradation of the DNA scaffold but should also consider residual histones, granular enzymes, and adaptive immune responses. Whether alveolar macrophages can autonomously release extracellular traps during IRI also remains to be established by direct evidence.

### 3.4. NETs and Renal IRI

Renal IRI is one of the mechanisms associated with acute kidney injury (AKI) and delayed graft function after kidney transplantation. Hypoxia and reperfusion can impair renal excretory and endocrine functions and are accompanied by peritubular capillary perfusion disturbances and no-reflow [182,183]. Clinical studies have reported elevated peripheral blood NET-associated markers during the early postoperative period in kidney transplant recipients, together with changes in lipid peroxidation products such as malondialdehyde (MDA), suggesting that NETs may participate in the early phase of renal IRI [19,108]. Within the renal microenvironment, products released from necrotic cells and metabolic reprogramming can both contribute to increased extracellular NET burden. Free DNA released from necrotic renal tubular epithelial cells can activate surrounding platelets and enhance platelet–neutrophil interactions, thereby inducing NET formation and release [109]. The ischemic microenvironment can also alter mitochondrial dynamics and metabolic programs in neutrophils [184,185,186]. Through purinergic receptor P2X1 (P2RX1) signaling, adenosine triphosphate (ATP) released from activated platelets can shift neutrophil metabolism toward glycolysis, providing metabolic support for NET formation [110]. In addition, complement component 3a (C3a)/C3a receptor (C3aR)-associated signaling can promote neutrophil chemotaxis and ROS accumulation and amplify local sterile inflammation [111,112].

These upstream events can increase the local NET burden in the kidney and extend associated injury from the peritubular microcirculation to renal tubules and, potentially, distant organs. Locally, NETs and NET-associated proteins such as Y-box binding protein 1 (YB-1) can injure proximal tubular epithelial cells (TECs) [113]. Double-stranded RNA (dsRNA) within NETs can activate TLR3 on TECs and induce PANoptosis, a process that may contribute to the progression of early AKI toward chronic kidney disease and fibrosis [187,188,189]. Renal IRI-associated NETs and their components may also contribute to distant-organ injury through the circulation. For example, circulating free histones and NET fragments can act on the pulmonary microvasculature and promote acute lung injury, suggesting a kidney–lung axis [9]. Systemic stress induced by local renal ischemia can also increase intestinal barrier permeability and promote microbial translocation; the resulting TLR4/Dectin-1-related signals can enhance NET release at the systemic level [114]. Furthermore, in models with an autoimmune background, ischemia-induced NETs can exacerbate lupus nephritis phenotypes through SYK-related signaling [115].

Based on evidence implicating NETs in peritubular inflammation, tubular injury, and distant complications, multilevel interventions have been investigated in models of renal IRI. At the level of upstream activation, the CXCL1/CXCL8 receptor antagonist G31P and the nuclear factor κB (NF-κB) inhibitor Ro 106-9920 can reduce neutrophil recruitment to the kidney and associated nuclear translocation events [116,117]. Targeting intracellular processes preceding NET release, the PAD4 inhibitor YW3-56 and the neutrophil elastase inhibitor sivelestat sodium can reduce chromatin decondensation, NET release, and MPO activity and improve renal functional indices in animal models [12,118]. Targeting released components, rTM can bind HMGB1 and has been shown to reduce free histones and NET-associated markers, attenuating vascular leakage and kidney–lung remote-organ injury [119,190,191]. For kidney transplant recipients receiving immunosuppressive therapy, future translational studies will need to evaluate the trade-offs among lesion-selective delivery, the risk of AKI-to-CKD progression, and preservation of antimicrobial host defense.

### 3.5. NETs and Cerebral IRI

Cerebral IRI can occur during reperfusion after intravenous thrombolysis or endovascular thrombectomy (EVT) in patients with acute ischemic stroke (AIS) and is associated with secondary brain injury and unfavorable outcomes. Restoration of cerebral blood flow can be accompanied by oxidative stress, disruption of calcium homeostasis, increased blood–brain barrier (BBB) permeability, and neurovascular-unit dysfunction [192]. Within this neuroimmune response, neutrophil infiltration and increased NET release may contribute to early cerebral IRI [193,194]. Relevant models have shown that NETs can accumulate within the microvasculature of the ipsilateral hemisphere after reperfusion and, through mechanisms involving VWF and platelet TLR4, form adhesive scaffolds that capture erythrocytes and extracellular vesicles, thereby promoting microvascular occlusion and no-reflow [120,121,195]. NET-rich microthrombi may also exhibit reduced sensitivity to tissue plasminogen activator (t-PA) and increase the risk of hemorrhagic transformation within ischemic brain tissue [122,123]. Upstream, plasma- or platelet-derived HMGB1 and ferroptosis accompanied by increased ROS in the context of endothelial ferroportin 1 (FPN1) deficiency have both been reported to promote NET release by neutrophils [124].

As BBB permeability increases, intravascular NETs and their associated components can enter the brain parenchyma and interact with glial cells. Microglia contribute to the clearance of extracellular debris; however, upregulation of NOD-like receptor X1 (NLRX1) in the cerebral IRI microenvironment can affect lysosomal function through mechanistic target of rapamycin (mTOR)/transcription factor EB (TFEB) signaling and is accompanied by increased galectin-3 expression and impaired phagocytic capacity. Insufficient clearance may allow NETs to persist within the brain parenchyma and contribute to neuronal injury [125,196]. Upregulation of FK506-binding protein 5 (FKBP5) can also promote a pro-inflammatory microglial phenotype and increase neutrophil NET formation and release through the C-C motif chemokine ligand 5 (CCL5)/MAPK axis, establishing a mutually reinforcing feedback loop between glial cells and NETs [126]. Interventions targeting this process have shown neuroprotective effects in animal models. Prophylactic administration of all-trans retinoic acid (atRA), which modulates the suppressor of cytokine signaling 1 (SOCS1)/signal transducer and activator of transcription 1 (STAT1) pathway, or early exercise accompanied by downregulation of neutrophil matrix metalloproteinase 25 (MMP25) has been reported to promote an N2-like neutrophil phenotype, enhance macrophage or microglial clearance of extracellular NETs, and increase the expression of tight-junction proteins (TJPs) [127,128].

Given the interrelationship among microthrombosis, BBB injury, and neuroimmune interactions, interventions targeting NET-associated processes in cerebral IRI are undergoing preclinical evaluation. Recombinant DNase I combined with t-PA can degrade the DNA scaffold of thrombi and improve thrombolysis-related outcomes in experimental studies [123]. Heparin, neonatal NET-inhibitory factor (nNIF), ivermectin, and Taohong Siwu decoction, the latter acting in association with the STAT1/NOD-, LRR- and pyrin domain-containing protein 3 (NLRP3)/GSDMD axis, have also shown neuroprotective effects in specific models [122,129,130,131]. However, the restricted permeability of the BBB to macromolecular therapeutics remains an important consideration for interventions targeting the central nervous system. To address this limitation, investigators have developed lipid nanoparticles (LNPs) that exploit the lesion-homing capacity of neutrophils to deliver cathepsin C inhibitors across the BBB to ischemic brain tissue [132]. In response to the oxidative and inflammatory environment following reperfusion, ROS-responsive biomimetic carriers have also been used to deliver PAD4 inhibitors such as GSK484 or Cl-amidine [18,133,197]. In relevant models, these carriers preferentially accumulate in microthrombi or regions with high ROS levels and are associated with reduced NET formation and cGAS–STING signaling, together with an increase in anti-inflammatory microglial phenotypes [134]. Future studies should evaluate the pharmacokinetics and safety of these delivery systems when combined with EVT or t-PA, as well as their effects on both large-vessel recanalization and microcirculatory endpoints.

### 3.6. NETs and Intestinal IRI

Intestinal IRI can occur in acute mesenteric ischemia, traumatic hemorrhagic shock, and extensive burns and may lead to intestinal tissue necrosis, systemic inflammatory response syndrome (SIRS), and multiple organ dysfunction syndrome (MODS) [198,199,200]. Intravital microscopy studies have shown that during the early phase of mesenteric ischemia, protease-activated receptor 1 (PAR1) on endothelial cells can mediate leukocyte adhesion, accompanied by NET formation and extracellular NET accumulation [135]. Within the intestinal microecological environment, the effects of NETs are context-dependent. During secondary gut microbial translocation or sepsis, NETs may limit bacterial dissemination; conversely, in predominantly ischemic models such as intestinal volvulus, excessive NET accumulation can promote microcirculatory thrombosis and is associated with increased mortality [136]. The commensal microbiota can also regulate this process, with certain beneficial microorganisms limiting ischemia-induced NET formation by attenuating neutrophil TLR4/TIR-domain-containing adapter-inducing interferon-β (TRIF) signaling [137].

Following these early microcirculatory alterations, cell-free DNA (cfDNA) and NETs can disrupt epithelial tight junctions, the cytoskeleton, and the vascular barrier [138]. Mechanistic studies suggest that NET accumulation can suppress FUNDC1-dependent mitophagy in intestinal epithelial cells and promote epithelial necroptosis through the TLR4/RIPK3 pathway [139]. PAD4-dependent NET formation can also induce ferroptosis in intestinal microvascular endothelial cells, further compromising vascular barrier integrity [140]. After local barrier disruption, gut-derived inflammatory mediators can enter the circulation and affect distant organs. HMGB1 and free histones released from necrotic intestinal epithelial cells can reach the lungs and promote pulmonary NET formation and release through MYD88-related signaling, thereby contributing to acute lung injury (ALI) [141]. Metabolic regulators such as the G protein-coupled receptor 81 (GPR81) agonist 3,5-dihydroxybenzoic acid (DHBA) can further influence this gut–lung axis and are accompanied by increased pulmonary levels of CitH3 and MPO–double-stranded DNA (dsDNA) complexes [142,201,202]. Circulating free histones and NETs may also contribute to distant hepatic injury through a gut–liver axis [143].

Given the potential extension of local barrier injury to distant organs, regulation of NET formation, release, and extracellular clearance has emerged as a candidate therapeutic strategy for intestinal IRI. Compared with antithrombotic therapies such as low-molecular-weight heparin or t-PA, exogenous DNase I can degrade NETs within the intestinal microenvironment, improve tight-junction protein expression, and attenuate oxidative stress in relevant animal models, without a reported increase in severe gastrointestinal bleeding [138,144]. However, the limited stability of DNase I in the circulation may restrict its in vivo application [203]. To address this limitation, investigators developed a conjugate of stroke-homing peptide (SHp) and DNase I, termed SHp–DNase I. This formulation exhibits greater stability in blood and preferentially accumulates within the ischemic and hypoxic intestinal microcirculation; in animal models, its administration has been associated with reduced NET burden and mortality [145]. In addition, genetic deletion of Padi4 or pharmacological inhibition of PAD4 to preserve mitophagy, use of rTM to neutralize circulating histones and mitigate distant hepatic injury, and administration of natural products such as Ganoderma lucidum polysaccharide peptide (GLPP) have all been evaluated in preclinical models [139,140,143,146].

### 3.7. NETs and Limb IRI

Limb IRI is a clinical complication that may occur after revascularization procedures, such as thrombectomy or bypass surgery, in patients with traumatic muscle ischemia or arterial thrombotic occlusion. Reperfusion can induce local edema and microvascular occlusion and, with disease progression, may be accompanied by myofiber necrosis, tissue fibrosis, and impaired limb motor function [204,205]. During the early phase of limb IRI, innate immune responses mediated by receptors including TLR4, TLR7, and TLR8 contribute to the initiation of inflammation [206,207]. The ischemic microenvironment can activate TLR4 signaling and downstream NF-κB, inducible nitric oxide synthase (iNOS), and poly(ADP-ribose) polymerase (PARP) expression, promote monocyte and neutrophil migration into perivascular and interstitial tissues, and increase NET formation and extracellular NET accumulation. In relevant models, TLR4 deficiency attenuates these immune responses and reduces both NET release and myofiber injury [147].

During the acute phase of injury, TLR-associated inflammatory signaling can coexist with the prothrombotic effects of NETs. NETs can provide a thrombotic scaffold and are associated with elevations in coagulation markers such as thrombin–antithrombin III complexes. Experimental interventions that degrade NET scaffolds or reduce neutrophil abundance do not exert identical effects on the microvasculature and parenchymal tissue. DNase I can reduce intravascular NETs and improve tissue perfusion and local blood flow in post-ischemic limbs; however, degradation of the extracellular DNA scaffold alone does not fully restore myofiber injury or intracellular ATP levels [147,148]. This observation suggests that, in addition to physical obstruction, NET-associated protein components may contribute to myofiber damage. In corresponding models, combined administration of DNase I and the neutrophil elastase inhibitor sivelestat has been associated with reduced NET formation, improved microcirculation and angiogenesis, and attenuation of early muscle injury [149].

During the recovery phase, skeletal muscle fibrosis represents a major long-term consequence of limb IRI, and NETs may participate in this remodeling process. Under sustained ischemic and inflammatory signaling, NETs can promote the proliferation of fibro-adipogenic progenitors (FAPs) and enhance ERK1/2 phosphorylation in injured tissues, thereby impairing muscle regenerative capacity and increasing fibrotic scar formation. Hydroxychloroquine, an inhibitor of TLR7/8/9 signaling, has been evaluated preclinically in this setting [208,209]. In relevant models, HCQ reduces NET release and alterations in inflammatory mediators such as interleukin-10 (IL-10) and tumor necrosis factor-α (TNF-α) while suppressing FAP proliferation and ERK1/2 signaling, thereby attenuating muscle fibrosis and improving myofiber regeneration [150]. Large-animal studies have also identified a relationship between NETs and extracellular-matrix remodeling. In a porcine limb IRI model, increased peptidylarginine deiminase (PAD) enzyme-associated protein citrullination, particularly elevated citrullinated fibrinogen, was associated with NET release and limb injury. PAD inhibition reduced related citrullination markers and NET formation and may preserve certain cellular functions [151]. Overall, current findings suggest that NET-targeted interventions may differentially affect acute microthrombosis and later muscle repair, although the optimal targets, therapeutic timing, and clinical effects remain to be established.

### 3.8. NETs and Skin IRI

Similar to limb IRI, cutaneous IRI originates from impaired peripheral tissue microcirculation, with its clinical consequences primarily involving skin-flap survival and wound repair. Cutaneous IRI can occur in surgical flap transplantation, pressure ulcers, and ischemic ulcers associated with Raynaud phenomenon. Ischemic and reperfusion stress can induce endothelial injury, microthrombosis, and tissue edema and is accompanied by an increase in pro-inflammatory macrophage phenotypes and leukocyte infiltration [210,211,212]. Under conditions such as freeze–thaw cycles induced by cryoablation or surgical vascular occlusion, local oxidative stress can activate neutrophils and increase NET release within the dermis and subcutaneous tissues [152].

Against this background of acute microcirculatory disturbance, NET accumulation in cutaneous tissues can be associated with flap necrosis, impaired angiogenesis, and delayed wound healing [213,214]. This process is influenced by endogenous immunoregulatory mechanisms. For example, the interleukin-36 receptor antagonist (IL-36Ra), encoded by Il36rn in mice, can limit neutrophil recruitment and contribute to the maintenance of tissue immune homeostasis [215]. In Il36rn-deficient mouse models, cutaneous IRI results in increased inflammatory-cell infiltration, with persistent NET accumulation observed from 4 to 72 h after reperfusion. Persisting extracellular DNA and associated proteins can further disrupt the dermal repair microenvironment and prolong wound healing [153].

Based on evidence involving both acute necrosis and delayed repair, clearance of NETs or neutralization of their associated components has been evaluated in models of cutaneous tissue repair. The small polyanionic compound mCBS can neutralize positively charged extracellular histones within NETs. Administration of mCBS within 5 min before or after the onset of cutaneous IRI can reduce histone-associated microvascular endothelial injury and increase the viable area of transplanted skin flaps in animal models [154]. Inhibition of NET formation has likewise shown tissue-protective effects. In an Il36rn-knockout mouse model of cutaneous IRI, the PAD inhibitor Cl-amidine reduced NET formation and inflammatory injury within affected tissues and improved indices of wound healing and tissue regeneration [153]. These findings support further evaluation of NET-targeted interventions in skin-flap transplantation and ischemic ulcers, although clinical studies are still required to establish their efficacy and safety. Taken together, evidence across different organs indicates that therapeutic interventions may target intracellular processes preceding NET release, extracellular NET scaffolds and associated components, or upstream cellular signaling pathways.

## 4. NET-Targeted Therapeutic Strategies for IRI

IRI is associated with perioperative organ dysfunction, early graft injury, and certain remote-organ complications. Across different organ systems, increased NET formation or release and insufficient clearance of extracellular NETs have repeatedly been associated with microvascular thrombosis, tissue barrier disruption, and amplification of inflammatory responses [8,193]. However, the temporal sequence, relative contribution, and maturity of evidence for these processes vary among organs, and the available therapeutic data are derived predominantly from organ-specific animal models. To delineate the mechanistic levels at which these interventions act, we categorize current strategies into degradation of extracellular NET scaffolds and associated components, inhibition of intracellular pathways preceding NET release, modulation of upstream sensing and intercellular signaling, promotion of inflammation resolution, and spatiotemporally targeted delivery. These strategies are summarized in Table 2.

### 4.1. Degradation and Neutralization of Extracellular NET Scaffolds and Associated Components

Cell-free DNA (cfDNA) and histones constitute major structural components of NETs and can contribute to cytotoxicity, coagulation, and inflammatory responses. Accordingly, degradation of the DNA scaffold or neutralization of associated protein components represents a downstream therapeutic approach that has been extensively explored in preclinical studies [16,220].

#### 4.1.1. DNase I and Modified DNase I Formulations

On the basis of its ability to cleave extracellular DNA scaffolds, exogenous DNase I has been evaluated in preclinical IRI models involving the intestine, lung, limb, and other organs and has been associated with reduced tissue injury [138,144,147,148,180]. In myocardial and cerebral ischemia models, DNase I administered alone or in combination with t-PA/rt-PA can reduce DNA scaffolds within microthrombi, facilitate thrombolysis, and improve endpoints such as microvascular obstruction and infarct size [17,123]. However, native free DNase I has a relatively short plasma half-life and can be affected by endogenous proteases [203]. Liu et al. developed SHp-DNase I by conjugating DNase I to a stroke-homing peptide, thereby achieving greater blood stability and preferential accumulation within the lesional microcirculation in experimental studies [145]. In addition, cationic polymers with high nucleic-acid affinity can function as macromolecular nucleic-acid scavengers by adsorbing cfDNA and NETs and may partially overcome the pharmacokinetic limitations of native enzyme preparations [221,222].

#### 4.1.2. Histone Neutralization and Disruption of Procoagulant Scaffolds

Unlike the DNA scaffold, NET-associated histones can directly contribute to increased microvascular permeability and remote-organ injury. Recombinant thrombomodulin (rTM) can modulate TLR4 signaling and bind circulating histones; in relevant models, its administration has been associated with attenuation of remote hepatic injury after intestinal IRI, reduced local ischemic necrosis in the liver, and improvement of kidney–lung-associated microvascular leakage [95,119,143,190,191]. Pretreatment with anti-histone immunoglobulin G (IgG) or administration of a neutralizing antibody against citrullinated histone H3 (hCitH3-mAb) has likewise been reported to reduce histone-associated toxicity and improve endpoints including remote-organ function and post-ischemic ventricular remodeling [9]. In cardiac IRI, recombinant human ADAMTS13 can cleave von Willebrand factor (VWF) incorporated into NET-associated thrombotic networks, reduce extracellular chromatin–thrombus complexes, and improve microcirculatory perfusion and cardiac contractile function [10].

### 4.2. Modulation of Intracellular Enzymatic and Signaling Pathways Preceding NET Release

In contrast to the clearance of already formed extracellular structures, modulation of intracellular enzymes and signaling pathways involved in chromatin decondensation, molecular mixing, and NET extrusion can intervene before extracellular NETs are generated [223].

#### 4.2.1. PAD4 Inhibition and Epigenetic Interference

PAD4 catalyzes histone citrullination and facilitates chromatin decondensation, acting as a regulatory enzyme in certain NET-forming pathways. In preclinical studies, genetic deletion of Padi4 or pharmacological reduction of PAD4 activity can decrease NET release and has been associated with attenuation of intestinal microvascular endothelial ferroptosis and cardiomyocyte apoptosis [83,139,140]. Pharmacological studies have employed the pan-PAD inhibitor Cl-amidine and the PAD4-targeting compound YW3-56, with improvements in tissue-injury indices observed in renal, cutaneous, and cerebral IRI models [12,18,126,153,197]. The second-generation PAD inhibitor BB-Cl-amidine has also demonstrated endothelial barrier-protective and NET-suppressive effects in microvascular models related to autoimmune disease [224].

#### 4.2.2. Inhibition of NE, NOX Activity, and Metabolic/Oxidative Stress Pathways

Because NET formation induced by different stimuli is not uniformly dependent on PAD4, NE nuclear translocation and oxidative and metabolic pathways represent additional therapeutic targets. The neutrophil elastase inhibitor sivelestat sodium can reduce MPO activity and CitH3 levels and has been shown in relevant models to attenuate renal tubular necrosis, improve hindlimb perfusion, and reduce muscle fibrosis [118,149]. The NOX inhibitor diphenyleneiodonium (DPI) can suppress ROS-dependent NET formation [86,99]. In drug-repurposing studies, allopurinol reduces ROS generation by inhibiting xanthine oxidase [86]. All-trans retinoic acid (atRA) and vitamin C combined with deferoxamine (DFOM) can also modulate intracellular redox homeostasis and, in cerebral ischemic preconditioning models, reduce NET-associated markers and neurological injury [124,127]. Alterations in cellular metabolism can likewise influence NET formation. Pyruvate kinase M2 (PKM2), a glycolytic enzyme, has been reported not only to regulate oxidative stress but also to function as a nuclear transcriptional coactivator that promotes inflammatory gene expression and NET formation [93,216]. Thus, PKM2-associated glycolytic signaling represents a candidate mechanistic link between metabolic reprogramming and NET-associated inflammation.

#### 4.2.3. Targeting GSDMD Pore Formation, the NLRP3 Inflammasome, and Related Intracellular Pathways

In certain models of lytic NETotic cell death, GSDMD-mediated membrane pore formation contributes to chromatin extrusion [225]. Disulfiram can inhibit GSDMD pore formation, and one study of lung transplantation-associated IRI further found that its administration was associated with suppression of mtDNA–cGAS–STING signaling and reductions in NET-associated indices [103]. Ivermectin has also been reported to reduce NET formation and release through related signaling pathways, whereas the ALOX12 inhibitor ML355 and the SYK inhibitor GS-9973 act on distinct upstream pathways [93,104,115,131]. Natural products or traditional compound formulations, including curcumin, tetramethylpyrazine (TMP), Taohong Siwu decoction (THSWD), formononetin (FMN), and Ganoderma lucidum polysaccharide peptide (GLPP), have been reported in preclinical studies to regulate pathways such as NLRP3/GSDMD and MAPK/ERK and thereby reduce NET release during reperfusion; some of these interventions have shown additive effects when combined with low-dose DNase I [77,98,99,130,146]. These findings require further evaluation with respect to compositional standardization, dose–response relationships, and independent reproducibility.

### 4.3. Modulation of Upstream Receptor Sensing and Intercellular Inflammatory/Procoagulant Signaling

When intracellular execution pathways are redundant, targeting initiating signals and intercellular communication may provide an alternative level of intervention. Increased NET formation and extracellular NET accumulation commonly arise from the combined effects of DAMPs and inflammatory mediators; therefore, antagonizing upstream sensing receptors or modulating signaling between heterogeneous cell populations may alter the local immune response within injured organs [11,226].

#### 4.3.1. Targeting DAMP–TLR Sensing, Complement Cascades, and Chemotactic Signaling

TLR4 can sense DAMPs such as HMGB1 during the early phase of IRI and contribute to neutrophil activation. Genetic deletion of Tlr4 or pharmacological inhibition of TLR4 has been shown in hepatic and pulmonary models to reduce NET formation and may also diminish NET-associated tumor-cell capture and pulmonary metastasis [86,94,181]. Hydroxychloroquine can antagonize endosomal TLR7/8/9 signaling and downregulate PAD4 expression, thereby attenuating hepatic IRI and ischemic limb muscle fibrosis in relevant models [96,150]. At the level of chemotactic signaling, the CXCL1/CXCL8 receptor antagonist G31P can reduce neutrophil recruitment to the ischemic kidney [117]. The IL-6 receptor blocker tocilizumab has been shown to reduce NET formation and infarct size in myocardial IRI models [80]. IL-1 receptor blockade has likewise been associated with attenuation of NET-related secondary tissue injury [227,228]. Complement signaling and NET formation can mutually reinforce one another. C5a and its receptor promote neutrophil chemotaxis and NET formation; in renal IRI models, C5 or C5aR antagonists can attenuate this feedback and reduce inflammatory injury [229,230]. Another study reported that disrupting the interaction between NET-derived double-stranded RNA (dsRNA) and TLR3 reduced PANoptosis of proximal tubular epithelial cells after renal ischemia, providing experimental support for receptor-level intervention [9].

#### 4.3.2. Modulating the Thromboinflammatory Axis and Platelet/Endothelial Interactions

Beyond DAMP and receptor signaling, platelet activation and endothelial barrier dysfunction within the ischemic microvasculature can promote NET release and contribute to reciprocal amplification between thrombosis and inflammation. Clopidogrel-mediated platelet inhibition or perioperative heparin administration in ischemic stroke may attenuate these intercellular signals, although their specific benefits and bleeding risks must be evaluated within the relevant clinical context [109,129]. In addition to inhibiting platelet aggregation, aspirin can reduce neutrophil migration toward ischemic lesions and attenuate NET-associated intravascular trapping effects [231,232]. Simvastatin can improve endothelial function and reduce IRI in hyperlipidemia-associated marginal donor liver models [97]. Colchicine can reduce microvascular obstruction after myocardial ischemia by modulating S100A8/A9-associated inflammatory pathways [79]. The endogenous peptide neonatal NET-inhibitory factor (nNIF) has also shown inhibitory effects on NET formation and microthrombosis in models of cerebral ischemia [122].

#### 4.3.3. Promoting Macrophage-Mediated Clearance and Resolution of Inflammation

Even when the generation of new NETs is reduced, extracellular NET structures and inflammatory debris already present within tissues still require clearance. Therefore, in addition to suppressing NET formation, release, and upstream activation, enhancing the phagocytic clearance of extracellular NETs and dying neutrophils by resident immune cells may facilitate resolution of inflammation [233]. Pro-resolving mediators such as resolvin D1 can modulate macrophage phenotypes and, in relevant models, promote the clearance of NETs and inflammatory debris while improving IRI-related outcomes in organs including the liver [217].

### 4.4. Spatiotemporally Targeted Delivery and Microenvironmental Modulation

Systemic drug administration may be limited by physical barriers such as the BBB and by the immunological risks associated with non-selective inhibition of NET formation, including an increased susceptibility to opportunistic infection. Accordingly, lesion-selective delivery and microenvironmental modulation based on materials science represent emerging strategies to improve therapeutic precision [234].

#### 4.4.1. Responsive Nanodelivery Systems and Lesion Targeting

To address limitations in drug accessibility and safety, Cheng et al. developed biomimetic lipid nanoparticles (LNPs) with neutrophil membrane-like properties to deliver the cathepsin C inhibitor brensocatib. In animal models, this system crossed the BBB and accumulated within ischemic brain regions, accompanied by improvements in indices of neurological injury [132]. ROS-responsive nanodelivery platforms have also been used to release agents such as GSK484 or Cl-amidine in the vicinity of microthrombi while simultaneously scavenging local ROS, modulating microglial phenotypes, and reducing NET-associated indices [133,134]. Jiang et al. exploited the lesion-homing and phagocytic properties of neutrophils to deliver nanoparticles carrying a sarco/endoplasmic reticulum Ca^2+^-ATPase (SERCA) activator to injured myocardium, resulting in attenuated cardiac remodeling in experimental models [85]. Inorganic nanomaterials with enzyme-mimetic activity have likewise been investigated. For example, platelet membrane-coated Prussian blue nanoparticles can preferentially accumulate in ischemic myocardium and have been associated with reduced local ROS levels, improved mitochondrial homeostasis, decreased NET formation, and lower levels of multiple cell-death markers [218,219]. These findings support further investigation of multitarget nanodelivery strategies; however, their biodistribution, long-term safety, manufacturing consistency, and clinical scalability remain to be established.

#### 4.4.2. Metabolic Remodeling of the Microenvironment and Non-Pharmacological Ecological Interventions

Beyond engineered delivery systems and pharmacological interventions, modulation of local metabolism, immune-cell states, or the tissue microbiological environment may also influence NET-associated injury. Early regular exercise can reduce NET release or extracellular NET accumulation in hepatic and cerebral ischemia models by downregulating neutrophil-surface MMP25 and reducing tissue adhesion, although the tissue-specific endpoints assessed in these two settings differ [100,128]. MSC-derived exosomes and gut microbiota-based interventions have also demonstrated anti-inflammatory or tissue-protective effects in selected models [76,78]. These studies provide clues for adjunctive strategies beyond direct pharmacological intervention; however, their mechanisms of action, organ-specific applicability, therapeutic windows, and immunological safety require independent validation within the corresponding IRI settings.

## 5. Limitations

Although NET-targeted interventions have demonstrated tissue-protective effects in preclinical models of IRI across multiple organs, several limitations continue to hinder translation from mechanistic studies to clinical application. First, current therapeutic approaches involve an inherent balance between potential benefit and risk. NETs contribute to host defense, and systemic inhibition of PAD4 or other neutrophil effector pathways may increase susceptibility to infection, warranting particular caution in immunocompromised populations such as organ transplant recipients. DNase I can cleave extracellular DNA scaffolds; however, residual histones and granular enzymes generated after NET degradation may retain cytotoxic or pro-inflammatory activity, suggesting that scaffold degradation and neutralization of toxic NET-associated components may need to be considered in combination.

Second, organ-specific and temporal heterogeneity limits the generalizability of existing findings. Different organs vary in microvascular architecture, local cellular composition, tolerance to ischemia, and the pathways that trigger NET formation. Studies of remote injury involving the gut–lung, gut–liver, and kidney–lung axes suggest the possibility of inter-organ interactions; however, the supporting evidence is derived predominantly from specific animal models and remains insufficient to establish a universally applicable cross-organ pathogenic network. Likewise, it cannot be assumed that local intervention in a single organ will necessarily prevent remote-organ injury. Most candidate therapies have been validated in only one organ, under a single dosing regimen, or at a limited number of time points, with insufficient cross-model replication, head-to-head comparison, or organ-specific pharmacokinetic evaluation.

Third, IRI is highly time-dependent, whereas many studies examine only one or a few observation points and may therefore fail to capture the dynamic changes in NET-associated processes across the ischemic phase, acute reperfusion phase, and subsequent repair phase. Clinically, there is also a lack of specific dynamic biomarkers capable of distinguishing ongoing NET formation, extracellular NET burden, and NET clearance status, making it difficult to define pathological thresholds and optimal therapeutic windows. Mechanistically, existing studies frequently infer NET formation from endpoint markers such as extracellular DNA, citrullinated histone H3 (CitH3), or MPO–DNA complexes, while rarely assessing plasma membrane integrity and irreversible cell death in parallel. Consequently, it remains difficult to distinguish lytic NETotic cell death from vital/non-lytic NET release. Whether exogenous interventions selectively affect one of these release modes, and the subsequent fate of membrane-intact cytoplasts within the microcirculation, have not yet been directly compared across organ-specific IRI settings.

Finally, the current understanding is based largely on young rodents without major comorbidities. Aging, metabolic syndrome, multimorbidity, and concomitant clinical medications may all alter neutrophil phenotypes and therapeutic responses. Future studies should therefore establish reproducible chains of evidence within clearly defined organ-specific and clinical contexts, separately delineate the temporal window of action, efficacy endpoints, and safety boundaries of candidate interventions, and avoid extrapolating findings from a single experimental model as universally applicable principles.

## 6. Conclusions and Future Directions

In summary, this review systematically summarizes the upstream triggers, local cellular interactions, major injurious effects, and interventional evidence related to NETs in IRI. Microthrombosis, endothelial or tissue barrier disruption, and amplification of inflammatory responses recur across studies involving multiple organs; however, the predominant signaling pathways, temporal dynamics, relevant effector cells, and clinical consequences differ among organ systems. For example, studies of cardiac and cerebral IRI have focused predominantly on microcirculatory obstruction and no-reflow, whereas hepatic studies additionally address the sinusoidal immune microenvironment and the risk of tumor recurrence. Pulmonary IRI research emphasizes interactions among endothelial cells, alveolar macrophages, and neutrophils, while renal and intestinal studies focus more specifically on tubular injury, barrier disruption, and selected remote-organ complications. Accordingly, the current evidence supports viewing NETs as recurrent but tissue context-dependent pathological contributors across different forms of IRI, rather than as components of a mechanistically uniform pathway shared by all organs.

Organ-specific and temporal heterogeneity also has important implications for clinical translation, and the balance between therapeutic benefit and potential risk should therefore be evaluated within each specific organ context. Systemic inhibition of neutrophil effector pathways such as PAD4 may attenuate sterile inflammation while simultaneously compromising antimicrobial host defense. Although DNase I can degrade the extracellular DNA scaffold, residual histones and granular enzymes may retain the capacity to induce secondary tissue injury. Drug accessibility, local NET burden, bleeding risk, immune status, and optimal therapeutic windows also differ substantially among organs. Consequently, doses, routes of administration, and efficacy endpoints established in one organ model cannot be directly extrapolated to another. The lack of organ-specific dynamic biomarkers and standardized methods for NET detection further limits patient stratification and assessment of therapeutic responses.

**Figure 3:**
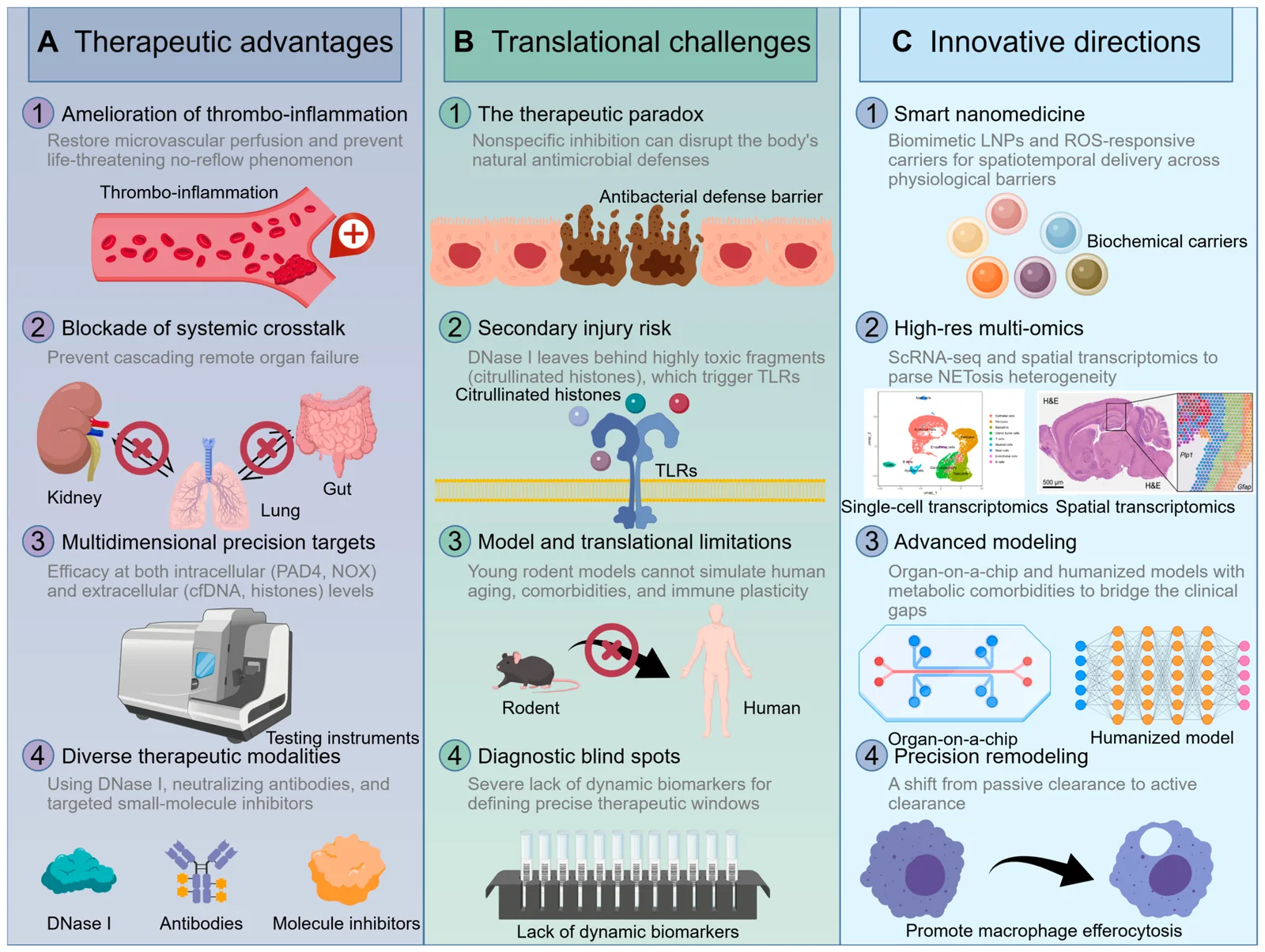
Therapeutic strategies targeting NET formation, release, and extracellular NET structures in IRI, together with translational challenges and future research directions. (**A**) Therapeutic advantages: PAD4 or NOX inhibitors modulate intracellular signaling preceding NET release, DNase I degrades the extracellular DNA scaffold, and neutralizing antibodies or other inhibitors target NET-associated effector proteins to attenuate thromboinflammation and microcirculatory dysfunction. (**B**) Translational challenges: non-selective inhibition may compromise host defense; DNase I does not simultaneously neutralize histones and granular proteases; and current experimental models, dynamic biomarkers, and therapeutic windows remain limited. (**C**) Innovative directions: development of tissue- and phase-specific delivery systems; integration of single-cell and spatial omics, NET-specific multiparametric detection, and more clinically relevant models; and strategies that promote NET clearance and resolution of inflammation.

To address these evidence gaps and improve both the mechanistic interpretability and translational potential of NET-targeted interventions, future research should advance along three major directions: organ-specific mechanistic investigation, longitudinal assessment, and lesion-targeted drug delivery. First, at the mechanistic level, live-cell imaging, simultaneous assessment of plasma membrane integrity and irreversible cell death, single-cell sequencing, and spatial and temporal omics should be integrated within individual organ-specific IRI models to define the relative contributions of nuclear- versus mitochondrial-derived extracellular NETs, as well as lytic NETotic cell death versus vital/non-lytic NET release. Pharmacological studies should also clearly define the level at which an intervention acts, distinguishing its effects on intracellular chromatin decondensation, NET extrusion, extracellular scaffold stability, associated protein components, or clearance processes, while determining whether neutrophil migration, phagocytosis, and antimicrobial functions are preserved. Second, in translational models and clinical evaluation, aging and metabolic comorbidity models, humanized systems, or organ-on-chip platforms should be incorporated according to the relevant organ and clinical scenario, together with longitudinal sampling at multiple time points to reduce bias arising from single-time-point observations. For remote complications such as the gut–lung and kidney–lung axes, for which experimental evidence already exists, causal relationships should be validated within clearly defined inter-organ contexts rather than generalized into a unified multiorgan network. In parallel, circulating or tissue biomarkers capable of distinguishing ongoing NET formation, extracellular NET burden, and clearance status should be developed to define pathological thresholds and therapeutic windows that are comparable across organs without necessarily being identical. Finally, drug-delivery strategies should be tailored to organ-specific barriers and lesion characteristics. For example, delivery routes and targeting carriers should be selected according to the blood–brain barrier, hepatic sinusoidal microcirculation, or the local environment of the transplanted lung, and may incorporate mechanism-based combinations of DNA scaffold degradation, neutralization of NET-associated proteins, ROS modulation, or enhancement of phagocytic clearance (Figure 3).

Therefore, the future of NET-targeted therapy for IRI should not center on establishing a single mechanistic model applicable to all organs, but rather on building clearly defined, reliably measurable, and reproducible evidence chains within specific organ contexts. Stratified investigation based on the organ-specific microenvironment, disease phase, and mode of NET release may help identify the predominant injury-associated processes and guide the selection of appropriate routes of administration, doses, and efficacy endpoints for candidate therapies. When combined with systematic evaluation of infection risk, bleeding, and immune homeostasis, validation in clinical samples and in experimental models that more closely reflect real-world comorbidity profiles will be essential for determining whether NET-targeted strategies can progress from experimental organ-protective approaches to clinically testable and stratified therapeutic interventions.

## Figures and Tables

**Table 1:** NET-associated biomarkers and molecular mechanisms regulating NET formation and release during IRI in various organs.

Organ IRI	NET-Associated Biomarkers	Inducers	Inhibitors	Molecular Mechanisms	Year	References
Heart	CitH3, Ly6G and plasma nucleosomes	\	rhADAMTS13	\	2014	[10]
	CitH3, MPO, MPO-DNA and PAD4	SPI1	\	SPI1/CST7/IFIT1/MMP9	2025	[73]
	CitH3 and MPO	Lactic acid	\	Lactic acid/MICU3/VDAC1	2025	[74]
	CitH3, MPO, MPO-DNA and NE	\	ALDH2	ALDH2/ERS/MGST2/LTC4/NOX2	2024	[75]
	CitH3, MPO, MPO-DNA and nucleosomes/histone-DNA	Gut microbiota	\	\	2022	[76]
	CitH3, MPO, NE and dsDNA	\	FMN	FMN/CD36/ERK5	2025	[77]
	MPO, histone H2B and DNA backbone	\	DNase I	\	2015	[17]
	CitH3, MPO, PAD4 and dsDNA	\	MSC-Exo	MSC-Exo/miR-199/NLRP3	2024	[78]
	CitH4, MPO and Ly6G	\	Colchicine	Colchicine/S100A8/A9	2025	[79]
	CitH3, MPO-DNA and dsDNA	\	Tocilizumab	\	2024	[80]
	CitH3, MPO-DNA, cfDNA and PAD4	\	4-OI	4-OI/ARID3A/THBS1/CD47/p38 MAPK	2025	[81]
	CitH3, MPO, PAD4 and dsDNA	TRAIL	\	TRAIL/DR5	2025	[82]
	CitH3 and MPO	\	GSK484	\	2025	[83]
	CitH3	\	hCitH3-mAb	\	2025	[84]
	Extracellular DNA	\	L-P-NP and SERCA	\	2025	[85]
Liver	CitH3, MPO-DNA, extracellular DNA and serum nucleosomes	Superoxide	Allopurinol and DPI	Superoxide/TLR4/NOX	2016	[86]
	CitH3, MPO-DNA and serum nucleosomes	\	DNase I	DNase I/DAMP/TLR	2015	[11]
	CitH3 and MPO-DNA	IL-33	\	IL-33/ST2	2017	[87]
	CitH3 and MPO	C5aR1	\	\	2024	[88]
	CitH3, MPO, and PAD4	TRIM21	\	TRIM21/SLC7A11	2025	[89]
	CitH3, MPO, PAD4 and extracellular DNA	\	CC1-L	CC1-L/S1P/S1PR2/S1PR3	2023	[90]
	CitH3, MPO, MPO-DNA, Ly6G and nucleosomes	\	HRG	\	2021	[91]
	CitH3, MPO, MPO-DNA, PAD4, NE and Ly6G	\	GSK-650394	GSK-650394/SGK1/IL-6/STAT3/SAA	2023	[92]
	CitH3, MPO, NE and Ly6G	\	GS-9973	GS-9973/SYK/PKM2/p-STAT3	2024	[93]
	CitH3 and MPO-DNA	TLR4	\	TLR4/ERK5-GPIIb/IIIa	2021	[94]
	CitH3, MPO, NE, extracellular DNA and Ly6G	Lipopolysaccharide	rTM	rTM/TLR4/ERK/JNK, rTM/TLR4/NADPH/ROS/PAD4	2022	[95]
	CitH3, PAD4 and cfDNA	CpG-ODN	HCQ	HCQ/TLR9/Rac2	2020	[96]
	CitH3, MPO, MPO-DNA and Ly6G	\	Simvastatin	Simvastatin/oxLDL/Mac-1	2024	[97]
	CitH3, MPO, MPO-DNA, extracellular DNA and Ly6G	\	Curcumin	Curcumin/MEK/ERK	2023	[98]
	CitH3, PAD4, extracellular DNA and Ly6G	\	TMP	TMP/NOX/ROS	2021	[99]
	CitH3 and MPO-DNA	\	Sports training	\	2021	[100]
Lung	CitH3, MPO, MPO-DNA, cfDNA and mtDNA	\	DNase I	DNase I/mtDNA/TLR9	2020	[101]
	CitH3, MPO, PAD4 and cfDNA	\	Cys A	Cys A/PPIF/SOCE/CN/NFAT	2024	[102]
	CitH3, MPO, MPO-DNA, mtDNA and dsDNA	\	Disulfiram	Disulfiram/GSDMD/cGAS-STING	2023	[103]
	CitH3, MPO, MPO-DNA, cfDNA and Ly6G	\	ML355	ML355/ALOX12–12-HETE/HMGB1/TLR4/MYD88	2024	[104]
	CitH3, MPO and dsDNA	EMAP-II	\	EMAP-II/PI3K/AKT/mtROS	2025	[105]
	CitH3, MPO, MPO-DNA, cfDNA and Ly6G	AM	\	AM/CTSC/p38 MAPK/NOX/ROS	2024	[106]
	PAD4 and extracellular DNA	\	DNase I	DNase I/TLR/MYD88	2019	[107]
Kidney	Circulating extracellular NET structures	MDA	\	\	2023	[108]
	Extracellular DNA	\	Clopidogrel	\	2017	[109]
	CitH3 and extracellular DNA	P2RX1	\	\	2021	[110]
	CitH3, MPO and extracellular DNA	\	Anti-Ly6G IgG	\	2022	[111]
	CitH3, MPO, PAD4, NE and extracellular DNA	\	Antioxidant	Antioxidant/C3a/C3aR/ROS	2023	[112]
	CitH3, MPO, NE and dsDNA	YB-1	\	\	2023	[113]
	CitH3, MPO-DNA, NE and extracellular histones	\	Anti-histone IgG	\	2017	[9]
	CitH3, MPO, dsDNA and Ly6G	Candida	\	Candida/ TLR4/dectin-1	2022	[114]
	CitH3, MPO, dsDNA and Ly6G	\	SYK inhibitor	SYK/TLR4/FcγR	2021	[115]
	CitH3 and MPO	\	Ro 106-9920	Ro 106-9920/NF-κB/MPO	2025	[116]
	NET-associated genes	\	G31P	G31P/CXCL1	2022	[117]
	CitH3, MPO, PAD4 and cfDNA	\	YW3-56	YW3-56/PAD4	2018	[12]
	dsDNA and histone H3	\	Sivelestat sodium	\	2025	[118]
	MPO and histones	\	rTM	\	2020	[119]
Brain	CitH3 and Ly6G	VWF	\	\	2023	[120]
	CitH3, elastase and NIMP-R14	\	DNase I	\	2022	[121]
	CitH3, MPO, MPO-DNA and NE	HMGB1	nNIF	\	2022	[122]
	Extracellular DNA and histones	\	DNase I and t-PA	\	2018	[123]
	CitH3	\	Vitamin C and DFOM	\	2025	[124]
	CitH3 and MPO	NLRX1	\	NLRX1/galectin-3/mTOR/TFEB	2025	[125]
	CitH3, MPO-DNA and dsDNA	FKBP5	Cl-amidine and AZD6244	FKBP5/CCL5/p38 MAPK	2025	[126]
	CitH3 and Ly6G	\	atRA	atRA/SOCS1/STAT1	2019	[127]
	CitH3 and MPO	\	Forced and voluntary exercise	MMP25/TJPs	2025	[128]
	CitH3, MPO-DNA, cfDNA and nucleosomes	\	Heparin	\	2025	[129]
	CitH3, MPO, NE, cfDNA and Ly6G	\	THSWD	THSWD/STAT1/NLRP3/GSDMD	2026	[130]
	CitH3 and MPO	\	IVM	\	2025	[131]
	CitH3 and MPO	\	LNP-AZD7986	AZD7986/DPP1	2025	[132]
	CitH3 and MPO	\	GSK484	GSK484/PAD4/ROS	2024	[133]
	CitH3 and NE	\	Cl-amidine	Cl-amidine/cGAS-STING/PAD4	2023	[134]
Gut	CitH3 and MPO	PAR1	\	\	2024	[135]
	NE and cfDNA	Lipopolysaccharide	\	\	2024	[136]
	CitH3 and MPO-DNA	\	Gut microbiota	Gut microbiota/TLR4/TRIF	2020	[137]
	CitH3, MPO, MPO-DNA and cfDNA	\	DNase I	\	2018	[138]
	CitH3-DNA and MPO-DNA	\	\	TLR4/RIPK3/FUNDC1	2024	[139]
	CitH3, CitH3-DNA and MPO-DNA	\	Ferrostatin-1, Urolithin A and AAV-Fundc1	\	2023	[140]
	CitH3, MPO-DNA and dsDNA	\	Anti-Ly6G IgG	Anti-Ly6G IgG/HMGB1/MYD88	2022	[141]
	CitH3, MPO-DNA and dsDNA	DHBA	\	DHBA/ GPR81/HMGB1	2023	[142]
	CitH3, Ly6G and extracellular histones	\	rTM	\	2019	[143]
	Extracellular DNA and histone-MPO	\	t-PA, LMWH and DNase I	\	2017	[144]
	CitH3, MPO and cfDNA	\	SHp-DNase I	\	2025	[145]
	CitH3, MPO, NE and cfDNA	\	GLPP	\	2023	[146]
Limb	MPO	\	Nuclease	Nuclease/TLR4/NF-κB	2013	[147]
	MPO	\	DNase I	\	2016	[148]
	MPO, NE and histone H3	\	DNase I and Sivelestat	\	2023	[149]
	CitH3 and MPO	\	HCQ	HCQ/TLR/ERK1/2	2020	[150]
	CitH4 and MPO	PAD4	\	\	2024	[151]
Skin	NE, extracellular DNA and histone H2B	Cryoablation	\	\	2025	[152]
	CitH3 and MPO	\	Cl-amidine	Cl-amidine/HMGB1/TLR4	2022	[153]
	cfDNA, histones and extracellular histones	\	mCBS	\	2020	[154]

**Table 2:** Therapeutic strategies targeting NET formation, NET release, or extracellular NETs in IRI across organs.

Treatment Strategies	Specific Targets	Representative Agents/Interventions	Regulatory Effects and Mechanisms	Research Phase	Organ IRI	References
Disassembly of extracellular NET scaffolds and neutralization of toxic NET components	Extracellular DNA/chromatin scaffold	DNase I	Cleave extracellular DNA/chromatin scaffolds, destabilize NET-associated microthrombi, and improve microvascular perfusion	Preclinical and early clinical studies	Heart	[17]
		DNase I			Lung	[180]
		DNase I			Brain	[123]
		DNase I and SHp-DNase I			Gut	[138,144,145]
		DNase I			Limb	[147,148]
	NET-associated histones and TLR4 signaling	hCitH3-mAb	Block TLR4 signaling, neutralize extracellular NET-associated histones, and alleviate distal-organ inflammatory crosstalk and microvascular leakage	Preclinical research and partial clinical application	Heart	[84]
		rTM			Liver	[95]
		rTM and antihistone IgG			Kidney	[9,119]
		rTM			Gut	[143]
	NET-associated procoagulant complexes	rhADAMTS13	Cleave NET-bound VWF and destabilize extracellular chromatin-thrombus complexes to restore perfusion	Preclinical study	Heart	[10]
Blockade of intracellular events preceding NET release	PAD4	GSK484	Inhibit histone hypercitrullination, block the core rate limiting step of chromatin depolymerization, and protect the endothelial barrier	Preclinical study	Heart	[83]
		YW3-56			Kidney	[12]
		Cl-amidine			Brain	[18,126,197]
		Padi4 KO			Gut	[139,140]
		Cl-amidine			Skin	[153]
	NE, NOX and metabolic enzymes	TMP, DPI, allopurinol and PKM2 inhibitors	Inhibit NE nuclear translocation and ROS production, suppress ROS-dependent NET formation and release, and reverse abnormal glycolysis and metabolic stress in immune cells	Preclinical research and clinical application	Liver	[86,93,99]
		Sivelestat sodium			Kidney	[118]
		atRA, vitamin C, DFOM and PKM2 inhibitors			Brain	[124,127,216]
		Sivelestat sodium			Limb	[149]
	GSDMD, NLRP3 and kinase	FMN	Inhibit GSDMD-mediated pore formation in the plasma membrane and negatively regulate the NLRP3 inflammasome and related intracellular kinase signaling pathways	Preclinical study	Heart	[77]
		GS-9973, curcumin, TMP and DPI			Liver	[93,98,99]
		Disulfiram and ML355			Lung	[103,104]
		GS-9973			Kidney	[115]
		Ivermectin and THSWD			Brain	[130,131]
		GLPP			Gut	[146]
Upstream receptor antagonism and blockade of multicellular inflammatory crosstalk	DAMPs TLR and chemokines	Tocilizumab	Cut off the early activation of neutrophils by microenvironmental DAMPs, downregulate PAD4 expression, block directed chemotaxis and systemic cascade inflammatory storm	Preclinical study	Heart	[80]
		TlR4 KO and HCQ			Liver	[86,94,96]
		TLR4 inhibitors			Lung	[181]
		G31P and dsRNA-TLR3 antagonist			Kidney	[9,117]
		HCQ			Limb	[150]
	Platelet/endothelial cell crosstalk	Colchicine	Disrupt the thromboinflammatory axis, suppress NET release promoted by activated platelets or damaged endothelium, and reduce intravascular entrapment by extracellular NETs	Clinical application	Heart	[79]
		Simvastatin			Liver	[97]
		Clopidogrel			Kidney	[109]
		Heparin and nNIF			Brain	[122,129]
	Macrophage exocytosis and phagocytosis	Resolvin D1	Promote macrophage polarization toward an M2-like anti-inflammatory phenotype, accelerate clearance of extracellular NET fragments in damaged tissues, and facilitate inflammation resolution	Preclinical study	Liver	[217]
Frontier spatiotemporal targeted delivery and system physiological and ecological regulation	Intelligent targeted delivery of nanomaterials	SR-localized nanoparticles	Break through physiological barriers, achieve micro thrombus anchoring and drug spatiotemporal specific release, avoid systemic immune suppression side effects	Preclinical study	Heart	[85]
		LNP-brensocatib, GSK484 nanoparticles and liposome nanocarriers			Brain	[132,133,134]
	Multi mimetic enzymes and nanoenzymes	Prussian blue nanoparticles and enzymes	Remove ROS in situ, restore mitochondrial homeostasis, and concurrently inhibit NET formation/release and PANoptosis-associated pathways	Preclinical study	Heart	[218,219]
	Microecological reshaping of physiological systems	MSC exosomes and gut microbiota	Downregulation of MMP25 weakens tissue adhesion, cuts off the intestinal target organ pro-inflammatory network, and reshapes the body’s anti ischemic homeostasis from a macroscopic biological perspective	Basic and clinical auxiliary intervention research	Heart	[76,78]
		Exercise intervention			Liver	[100,128]

## Data Availability

No new data were created or analyzed in this study. Data sharing is not applicable to this article.

## Abbreviations

The following abbreviations are used in this manuscript:

4-OI	4-Octyl itaconate
12-HETE	12-Hydroxyeicosatetraenoic acid
AAV	Adeno-associated virus
ADAMTS13	A disintegrin and metalloproteinase with thrombospondin type 1 motif, member 13
AIS	Acute ischemic stroke
AKI	Acute kidney injury
AKT	Protein kinase B
ALDH2	Aldehyde dehydrogenase 2
ALI	Acute lung injury
ALOX12	Arachidonate 12-lipoxygenase
AM/AMs	Alveolar macrophage(s)
ARID3A	AT-rich interaction domain 3A
ATP	Adenosine triphosphate
atRA	All-trans retinoic acid
BBB	Blood–brain barrier
C3a	Complement component 3a
C3aR	Complement component 3a receptor
C5	Complement component 5
C5a	Complement component 5a
C5aR/C5aR1	Complement component 5a receptor 1
Ca^2+^	Calcium ion
CC1-L	Long isoform of carcinoembryonic antigen-related cell adhesion molecule 1
CCL5	C-C motif chemokine ligand 5
CD4	Cluster of differentiation 4
CD36	Cluster of differentiation 36
CD47	Cluster of differentiation 47
cfDNA	Cell-free DNA
cGAS	Cyclic GMP–AMP synthase
CitH3	Citrullinated histone H3
CitH4	Citrullinated histone H4
CN	Calcineurin
CpG-ODN	Cytosine-phosphate-guanine oligodeoxynucleotide
CST7	Cystatin F
CTCs	Circulating tumor cells
CTSC	Cathepsin C
CXCL1	C-X-C motif chemokine ligand 1
CXCL8	C-X-C motif chemokine ligand 8
Cys A	Cyclosporin A
DAMPs	Damage-associated molecular patterns
DFOM	Deferoxamine
DHBA	3,5-Dihydroxybenzoic acid
DNA	Deoxyribonucleic acid
DNase I	Deoxyribonuclease I
DPI	Diphenyleneiodonium
DPP1	Dipeptidyl peptidase 1
DR5	Death receptor 5
dsDNA	Double-stranded DNA
dsRNA	Double-stranded RNA
EMAP-II	Endothelial monocyte-activating polypeptide II
ERK	Extracellular signal-regulated kinase
ERK1/2	Extracellular signal-regulated kinases 1 and 2
ERK5	Extracellular signal-regulated kinase 5
ERS	Endoplasmic reticulum stress
EVs	Extracellular vesicles
EVT	Endovascular thrombectomy
FAPs	Fibro-adipogenic progenitors
FcRs	Fc receptors
FcγR	Fc gamma receptor
FKBP5	FK506-binding protein 5
FMN	Formononetin
FPN1	Ferroportin 1
Fundc1	FUN14 domain-containing 1 (mouse gene)
FUNDC1	FUN14 domain-containing protein 1
GLPP	Ganoderma lucidum polysaccharide peptide
GM-CSF	Granulocyte–macrophage colony-stimulating factor
GPCRs	G protein-coupled receptors
GPR81	G protein-coupled receptor 81
GSDMD	Gasdermin D
GSDMD-NT	Gasdermin D N-terminal fragment
hCitH3-mAb	Monoclonal antibody against citrullinated histone H3
HCQ	Hydroxychloroquine
HMGB1	High mobility group box 1
HRG	Histidine-rich glycoprotein
H_2_O_2_	Hydrogen peroxide
IFIT1	Interferon-induced protein with tetratricopeptide repeats 1
IgG	Immunoglobulin G
Il36rn	Interleukin 36 receptor antagonist (mouse gene)
IL-1	Interleukin-1
IL-1β	Interleukin-1 beta
IL-6	Interleukin-6
IL-10	Interleukin-10
IL-33	Interleukin-33
IL-36Ra	Interleukin-36 receptor antagonist
iNOS	Inducible nitric oxide synthase
IRI	Ischemia–reperfusion injury
IVM	Ivermectin
JNK	c-Jun N-terminal kinase
KO	Knockout
L-P-NPs	SERCA activator-loaded sarcoplasmic reticulum-localized nanoparticles
LMP	Lysosomal membrane permeabilization
LMWH	Low-molecular-weight heparin
LNP/LNPs	Lipid nanoparticle(s)
LPS	Lipopolysaccharide
LTC4	Leukotriene C4
Ly6G	Lymphocyte antigen 6 complex locus G6D
Mac-1	Macrophage-1 antigen
MAMs	Mitochondria-associated endoplasmic reticulum membranes
MAPK	Mitogen-activated protein kinase
mCBS	Methyl β-D-cellobioside per-O-sulfate
MDA	Malondialdehyde
MEK	Mitogen-activated protein kinase
MGST2	Microsomal glutathione S-transferase 2
MICU3	Mitochondrial calcium uptake family member 3
miR-199	MicroRNA-199
MMP9	Matrix metalloproteinase 9
MMP25	Matrix metalloproteinase 25
MODS	Multiple organ dysfunction syndrome
MPO	Myeloperoxidase
MPO–DNA	Myeloperoxidase–DNA complex
MSC-Exo	Mesenchymal stem cell-derived exosomes
MSC/MSCs	Mesenchymal stem cell(s)
mtDNA	Mitochondrial DNA
mTOR	Mechanistic target of rapamycin
mtROS	Mitochondrial reactive oxygen species
MYD88	Myeloid differentiation primary response 88
NADPH	Nicotinamide adenine dinucleotide phosphate
nDNA	Nuclear DNA
NE	Neutrophil elastase
NET/NETs	Neutrophil extracellular trap(s)
NF-κB	Nuclear factor kappa B
NFAT	Nuclear factor of activated T cells
NLRP3	NOD-, LRR- and pyrin domain-containing protein 3
NLRX1	NOD-like receptor X1
nNIF	Neonatal NET-inhibitory factor
NOX	Nicotinamide adenine dinucleotide phosphate oxidase
NOX2	Nicotinamide adenine dinucleotide phosphate oxidase 2
NOX4	Nicotinamide adenine dinucleotide phosphate oxidase 4
ORAI1	Calcium release-activated calcium modulator 1
oxLDL	Oxidized low-density lipoprotein
P2RX1	Purinergic receptor P2X1
PAD	Peptidylarginine deiminase
PAD4	Peptidylarginine deiminase 4
Padi4	Peptidylarginine deiminase 4 (mouse gene)
PAMPs	Pathogen-associated molecular patterns
PAR1	Protease-activated receptor 1
PARP	Poly(ADP-ribose) polymerase
PGD	Primary graft dysfunction
PI3K	Phosphoinositide 3-kinase
PKC	Protein kinase C
PKM2	Pyruvate kinase M2
PMA	Phorbol 12-myristate 13-acetate
PMN-MDSCs	Polymorphonuclear myeloid-derived suppressor cells
PPIF	Peptidyl-prolyl cis-trans isomerase F
PR3	Proteinase 3
PRRs	Pattern recognition receptors
rhADAMTS13	Recombinant human a disintegrin and metalloproteinase with thrombospondin type 1 motif, member 13
RIPK3	Receptor-interacting protein kinase 3
RNA	Ribonucleic acid
ROS	Reactive oxygen species
rt-PA	Recombinant tissue plasminogen activator
rTM	Recombinant thrombomodulin
S1P	Sphingosine-1-phosphate
S1PR2	Sphingosine-1-phosphate receptor 2
S1PR3	Sphingosine-1-phosphate receptor 3
S100A8	S100 calcium-binding protein A8
S100A9	S100 calcium-binding protein A9
SAA	Serum amyloid A
SERCA	Sarco/endoplasmic reticulum Ca^2+^-ATPase
SGK1	Serum/glucocorticoid-regulated kinase 1
SHp	Stroke-homing peptide
SIRS	Systemic inflammatory response syndrome
SLC7A11	Solute carrier family 7 member 11
SOCE	Store-operated calcium entry
SOCS1	Suppressor of cytokine signaling 1
SOD	Superoxide dismutase
SPI1	Spi-1 proto-oncogene, ETS transcription factor
SR	Sarcoplasmic reticulum
ST2	Suppression of tumorigenicity 2
STAT1	Signal transducer and activator of transcription 1
STAT3	Signal transducer and activator of transcription 3
STIM1	Stromal interaction molecule 1
STING	Stimulator of interferon genes
SYK	Spleen tyrosine kinase
t-PA	Tissue plasminogen activator
TECs	Tubular epithelial cells
TFEB	Transcription factor EB
THBS1	Thrombospondin 1
THSWD	Taohong Siwu decoction
TJPs	Tight-junction proteins
Tlr4	Toll-like receptor 4 (mouse gene)
TLR/TLRs	Toll-like receptor(s)
TMP	Tetramethylpyrazine
TNF-α	Tumor necrosis factor-alpha
TRAIL	Tumor necrosis factor-related apoptosis-inducing ligand
TRIF	TIR-domain-containing adapter-inducing interferon-β
TRIM21	Tripartite motif-containing 21
VDAC1	Voltage-dependent anion channel 1
VWF	Von Willebrand factor
YB-1	Y-box-binding protein 1
